# TRF2-RAP1 is required to protect telomeres from engaging in homologous recombination-mediated deletions and fusions

**DOI:** 10.1038/ncomms10881

**Published:** 2016-03-04

**Authors:** Rekha Rai, Yong Chen, Ming Lei, Sandy Chang

**Affiliations:** 1 Department of Laboratory Medicine, Yale University School of Medicine, 330 Cedar Street, New Haven, Connecticut 06520, USA; 2 National Center for Protein Science Shanghai, State Key Laboratory of Molecular Biology, Institute of Biochemistry and Cell Biology, Shanghai Institutes for Biological Sciences, Chinese Academy of Sciences 333, Haike Rd., Shanghai 201210, China; 3 Department of Pathology, Yale University School of Medicine, 330 Cedar Street, New Haven, Connecticut 06520, USA; 4 Department of Molecular Biophysics and Biochemistry, Yale University School of Medicine, 330 Cedar Street, New Haven, Connecticut 06520, USA

## Abstract

Repressor/activator protein 1 (RAP1) is a highly conserved telomere-interacting protein. Yeast Rap1 protects telomeres from non-homologous end joining (NHEJ), plays important roles in telomere length control and is involved in transcriptional gene regulation. However, a role for mammalian RAP1 in telomere end protection remains controversial. Here we present evidence that mammalian RAP1 is essential to protect telomere from homology directed repair (HDR) of telomeres. RAP1 cooperates with the basic domain of TRF2 (TRF2^B^) to repress PARP1 and SLX4 localization to telomeres. Without RAP1 and TRF2^B^, PARP1 and SLX4 HR factors promote rapid telomere resection, resulting in catastrophic telomere loss and the generation of telomere-free chromosome fusions in both mouse and human cells. The RAP1 Myb domain is required to repress both telomere loss and formation of telomere-free fusions. Our results highlight the importance of the RAP1-TRF2 heterodimer in protecting telomeres from inappropriate processing by the HDR pathway.

## Abstract (web summary)

While yeast Rap1 regulates telomere length and protects telomeres from non-homologous end joining, its role in higher eukaryotes is controversial. Here the authors present evidence that in mammals, RAP1 cooperates with TRF2 to prevent homologous recombination-mediated repair of telomeres.

## Graphical abstract

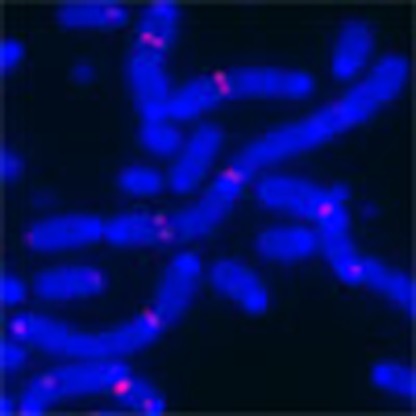

Mammalian telomeres are protein–DNA complexes that cap the ends of linear chromosomes. Maintenance of proper telomere functions requires both telomerase and the specialized six protein Shelterin complex that binds telomeres^1^,^2^. TTAGGG-repeat factor 1/2 (TRF1 and TRF2) interact with double-stranded telomere, and bind to the single-stranded (ss) telomere DNA-binding protein POT1 and its heterodimer TPP1 through TIN2. RAP1 (repressor/activator protein 1) is the most highly conserved Shelterin component and the only one that is conserved from yeast to mammals. All RAP1 proteins possess a N-terminal BRCT domain, one or two central Myb domain(s) and a C-terminal RCT domain^3^,^4^,^5^,^6^. The *Saccharomyces cerevisiae* Rap1 (ScRap1) was originally discovered as a transcriptional regulator and later shown to be a multifunctional protein, with roles in subtelomeric silencing, telomere length regulation and telomere end protection against non-homologous end-joining (NHEJ)-mediated DNA repair^7^,^8^,^9^,^10^,^11^. ScRap1 directly interact with telomeric DNA through its two Myb domains and regulates gene silencing by recruiting Sir3p and Sir4p to chromatin via its RCT domain, and also interacts with the Rap1-interacting factors Rif1 and Rif2 to regulate telomere length and end protective functions^3^,^6^,^7^,^8^,^9^,^10^,^12^. Like ScRap1, the *Schizosaccharomyces pombe* Rap1 (SpRap1) also has roles in telomere length regulation, telomere end protection and telomere silencing^13^,^14^. However, unlike ScRap1, SpRap1 cannot bind directly to telomeres. Instead, structural studies reveal that its localization to telomeres depends on the interaction between its C-terminus with Taz1, an orthologue of mammalian TRF1 and TRF2 (ref. 6).

Mammalian RAP1 shares certain functional similarities with its yeast counterparts. Mouse RAP1 interacts with both telomeric and subtelomeric DNA and plays a role in transcriptional control of metabolic genes involved in body weight control^15^,^16^,^17^,^18^. We have previously shown through structural-functional studies that like SpRap1, localization of mammalian RAP1 to telomeres requires interaction of its RCT domain with TRF2's RAP1-binding motif (RBM)^6^. In particular, mutating a leucine residue to an arginine in the TRF2^RBM^ domain abolished RAP1's interaction with TRF2 and its recruitment to telomeres^6^. Mouse telomeres with TRF2 but devoid of RAP1 are protected from end-to-end chromosomal fusions, indicating that RAP1 is dispensable for protection from NHEJ-mediated repair of telomeres^6^. This observation is further supported by *Rap1* knockout studies, in which *Rap1*^*−/−*^ mouse embryo fibroblasts (MEFs) do not display increased end-to-end chromosome fusions^16^,^19^. However, in MEFs expressing mutant TRF2 that is unable to interact with endogenous RAP1, we observed increased loss of telomeric signals, elevated telomere sister chromatid exchanges (T-SCEs) and increased chromosome fusions due to increased homologous recombination (HR) at telomeres. These results suggest that mouse RAP1 plays a role in protecting telomeres from initiating homology directed repair (HDR)^6^. In support of this notion, *Rap1*^*−/−*^ MEFs exhibit increased T-SCEs, suggesting that it functions to repress telomere HDR^19^.

In this report, we present strong evidence that mammalian RAP1 plays an important role in telomere end protection. RAP1 cooperates with the basic domain of TRF2 to repress PARP1 localization to telomeres, which in turn inhibits the Holiday junction (HJ) resolvase SLX4's telomeric localization. In the absence of RAP1 and TRF2^B^, PARP1, SLX4 and proteins involved in HDR promote rapid telomere resection, catastrophic telomere loss and formation of telomere-free chromosome ends, culminating in massive telomere-free chromosome fusions in both mouse and human cells. Our results thus highlight the importance of the RAP1-TRF2 heterodimer in protecting telomeres from inappropriate processing by the HDR pathway.

## Results

### TRF2^B^ cooperates with RAP1 to prevent telomere deletion

**Figure 1:**
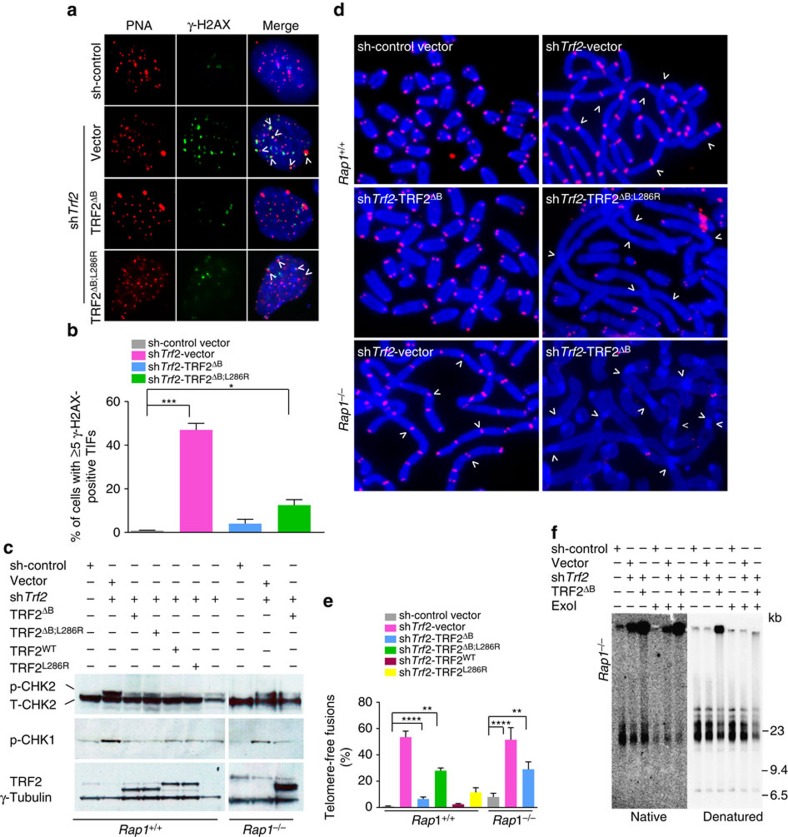
Mammalian RAP1 and TRF2^B^ prevent rapid telomere deletions and telomere-free fusions. (**a**) SV40^LT^-immortalized WT MEFs were reconstituted with TRF2^ΔB^ or TRF2^ΔB; L286R^ before *Trf2* shRNA was used to deplete endogenous TRF2. γ-H2AX-positive foci (green) at telomeres (red) was used to monitor the number of dysfunctional telomere induced foci (TIFs). 4,6-diamidino-2-phenylindole (DAPI) was used to stain nuclei (blue). (**b**) Quantification of percent of cells with ≥5 γ-H2AX-positive TIFs shown in **a**. Data represent the mean of two independent experiments±standard deviation (s.d.). A minimum of 100 nuclei were examined per experiment. *P<0.05, ***P<0.0003; one-way analysis of variance (ANOVA). (**c**) Immunoblots for TRF2, total (T) CHK2, phospho (p)-CHK2 and p-CHK1 in *Rap1*^*+/+*^ and *Rap1*^*−/−*^ MEFs reconstituted with the indicated DNAs. sh*Trf2* was used to deplete endogenous TRF2. γ-Tubulin was used as loading control. Note that our anti-TRF2 antibody was able to detect only the overexpressed TRF2 protein and not endogenous TRF2. (**d**) Metaphase spreads were prepared from *Rap1*^*+/+*^ cells expressing TRF2^ΔB; L286R^ or *Rap1*^*−/−*^ MEFs expressing TRF2^ΔB^, followed by removal of endogenous TRF2 with *Trf2* shRNA. Telomere fusions were visualized by telomere PNA-FISH (red) and DAPI (blue). Arrows point to a few of the chromosomes' fusion sites. Note that chromosome fusions lacking telomeric signals at fusion sites are a prominent feature of cells expressing TRF2^ΔB; L286R^. (**e**) Quantification of telomere fusions from images shown in **d**. Data are representative of the mean of three independent experiments±s.d. A minimum of 70 metaphases were examined per experiment (**P<0.001, ****P<0.0001; one-way ANOVA). (**f**) Genomic DNA from *Rap1*^*−/−*^ MEFs expressing the indicated cDNA constructs with or without *Exo*I treatment were fractionated with a clamped homogenous electric field (CHEF) gel. In-gel hybridization was performed using a radiolabelled (CCCTAA)_4_ probe to detect single-stranded telomere overhang under native conditions (left) and total telomeric repeats under denaturing conditions (right). Molecular weights are displayed on the right.

Using the TRF2^L286R^ separation of function mutation that cannot interact with endogenous mouse RAP1, we have shown previously that telomeres devoid of RAP1 display loss of telomeric signals and a slight increase in end-to-end chromosomal fusions^6^. In contrast to chromosome fusions in cells devoid of TRF2, in which nearly 100% of chromosome fusion sites contain prominent telomeric signals, chromosome fusions in the absence of RAP1 do not possess any telomeric signal at fusion sites, suggesting that near complete telomere attrition occurred at chromosome ends before fusion^6^. Given the relative paucity of fused chromosomes observed in cells expressing TRF2^L286R^, we hypothesized that RAP1 co-operates with other Shelterin components to repress HDR at telomeres. We focused on the N-terminal basic domain of TRF2 (TRF2^B^), as previous reports suggest that deletion of this domain promotes telomere shortening through HR-mediated telomere (t)-circle formation^20^,^21^. To examine whether RAP1 cooperates with TRF2^B^ for telomere end protection, we expressed either wild-type (WT) TRF2, TRF2^ΔB^, TRF2^L286R^ or TRF2^ΔB; L286R^ (ref. 6) in WT MEFs and then used sh*Trf2* to remove endogenous TRF2 (Supplementary Fig. 1a–c) (ref. 22). We have shown previously that reconstitution of WT TRF2 in this manner completely rescued the telomere dysfunctional phenotypes associated with *TRF2* depletion, including elimination of dysfunctional telomere-induced foci (TIF) formation as well as end-to-end chromosome fusions^22^. WT TRF2 and all mutant TRF2 constructs all localized to telomeres (Supplementary Fig. 1d). Compared with vector control, WT TRF2 and all mutant TRF2 constructs also protected telomere ends from activating a DNA damage response, as evidenced by the significantly reduced TIF formation and phosphorylation of the CHK1/2 kinases (Fig. 1a–c; uncropped western blots are provided in Supplementary Fig. 7). The number of chromosome fusions observed in WT MEFs expressing WT TRF2, TRF2^ΔB^ and TRF2^L286R^ never exceeded ∼12% over vector control. In sharp contrast, within 120 h of expressing TRF2^ΔB; L286R^, ∼53% of all chromosome ends display telomere-free ends, suggesting a rapid loss of telomeric signals at both leading and lagging chromosome ends (Supplementary Fig. 1h,i). In addition, end-to-end chromosome fusions involving ∼32% of all chromosome ends were observed, with fusion sites completely devoid of telomere signals (which we term telomere-free fusions; Fig. 1d,e and Supplementary Fig. 1e,f). Time-course analysis revealed that telomere-free chromosome ends were observed as early as 48 h after TRF2^ΔB; L286R^ expression in WT MEFs, and this phenotype always preceded the generation of telomere-free fusions (Supplementary Fig. 1f). To further confirm that RAP1 cooperates with the TRF2^B^ domain to repress rapid telomere loss and telomere-free fusions, we removed endogenous TRF2 in *Rap1*^*−/−*^ MEFs and reconstituted with TRF2^ΔB^ (ref. 15). We found that 46% of chromosome ends were completely devoid of telomeric signals, and telomere-free fusions identical to those observed in WT MEFs expressing TRF2^ΔB; L286R^ were present in 100% of metaphases, involving ∼30% of all chromosome ends scored (Fig. 1d,e and Supplementary Fig. 1h, 1j). TRF length analysis further confirmed marked telomere loss and increased ss G-overhang in both *Rap1*^*−/−*^ MEFs expressing TRF2^ΔB^ and in WT cells expressing TRF2^ΔB; L286R^ (Fig. 1f and Supplementary Fig. 1g, 1k,1l). Taken together, these results suggest that both RAP1 and the basic domain of TRF2 are required to repress rapid telomere deletion and telomere-free fusions.

### Telomere-free fusions are not due to C-NHEJ or A-NHEJ repair

**Figure 2:**
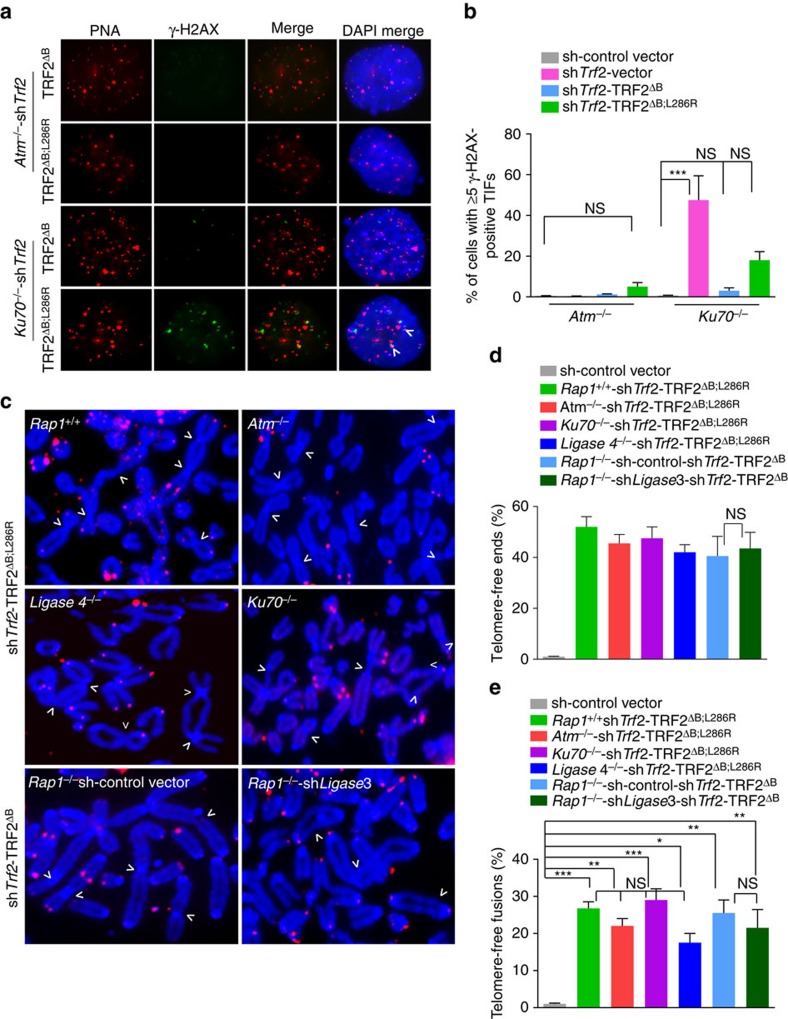
Telomere-free fusions are not due to C-NHEJ or A-NHEJ repair. (**a**) *ATM*^*−/−*^ and *Ku70*^*−/−*^ MEFs were reconstituted with TRF2^ΔB^ or TRF2^ΔB; L286R^ before shRNA-mediated depletion of endogenous TRF2. Telomeres were visualized with telomere PNA-FISH (red), anti-γ-H2AX antibody (green) was used to detect γ-H2AX and DAPI to stain nuclei (blue). (**b**) Quantification of percent of cells with ≥5 γ-H2AX-positive TIFs shown in **a**. Data are averaged from two independent experiments±s.d., and a minimum of 100 nuclei were examined per experiment. ***P<0.0001; one-way analysis of variance (ANOVA). NS, non-significant values. (**c**) *Rap1*^*+/+*^*, ATM*^*−/−*^. *Ligase 4*^*−/−*^*and Ku70*^*−/−*^ MEFs reconstituted with TRF2^ΔB; L286R^, and *Rap1*^*−/−*^ MEFs reconstituted with TRF2^ΔB^ and expressing either control vector or shRNA against *Ligase 3*, were treated with *Trf2* shRNA to deplete endogenous TRF2. Metaphase spreads were prepared and chromosome fusions visualized by telomere PNA-FISH (red) and DAPI (blue). Arrows point to fused chromosomes without telomeric signals at sites of fusion. (**d**) Quantification of telomere-free chromosome ends shown in **c**. Data represent the average of two experiments±s.d.; a minimum of 40 metaphases were examined per experiment. NS, non-significant values. (**e**) Quantification of telomere-free fusions shown in **c**. Data are the representative of the mean from three independent experiments ±s.d. from a minimum of 40 metaphases examined per experiment (*P<0.01, **P<0.001, ***P<0.0005; one-way ANOVA).

Telomeres devoid of TRF2-RAP1 or POT1-TPP1 are repaired via either the classical Ligase 4-mediated, non-homologous end-joining (C-NHEJ) or the Ligase 3-mediated, alternative non-homologous end-joining (A-NHEJ) DNA repair pathways, respectively^23^,^24^,^25^,^26^. To understand mechanistically how chromosome ends devoid of RAP1 and TRF2^B^ are repaired, we first expressed TRF2^ΔB; L286R^ in MEFs genetically deficient for factors required for C-NHEJ-mediated repair, including ATM, Ku70 and Ligase4. In *Atm*^*−/−*^ MEFs, knockdown of *Trf2* was unable to activate a DNA damage response, but the number of telomere-free chromosome ends and telomere-free fusions generated were indistinguishable from those observed in WT controls (Fig. 2a–e and Supplementary Fig. 2a). Compared with sh*Trf2*-treated WT MEFs, expression of TRF2^ΔB; L286R^ in *Ku70*^*−/−*^ and *Ligase4*^*−/−*^ MEFs reduced TIF formation and the activation of a CHK2-dependent DNA damage response, but telomere-free signals at chromosome ends and telomere-free fusions remain elevated (Fig. 2–e and Supplementary Fig. 2a). We also examined the requirement for Ligase 3 in the generation of these chromosomal aberrations. Treatment of *Rap1*^*−/−*^ MEFs expressing TRF2^ΔB^ with short hairpin RNA (shRNA) against *Ligase 3* did not reduce either the number of telomere-free chromosome ends or the number of telomere-free fusions (Fig. 2c–e and Supplementary Fig. 2b). These results indicate that the aberrant telomere phenotypes observed in cells devoid of RAP1 and TRF2^B^ are not due to activation of either the C-NHEJ- or A-NHEJ-mediated DNA repair pathways.

### HDR rapidly removes telomeres lacking RAP1-TRF2^B^

**Figure 3:**
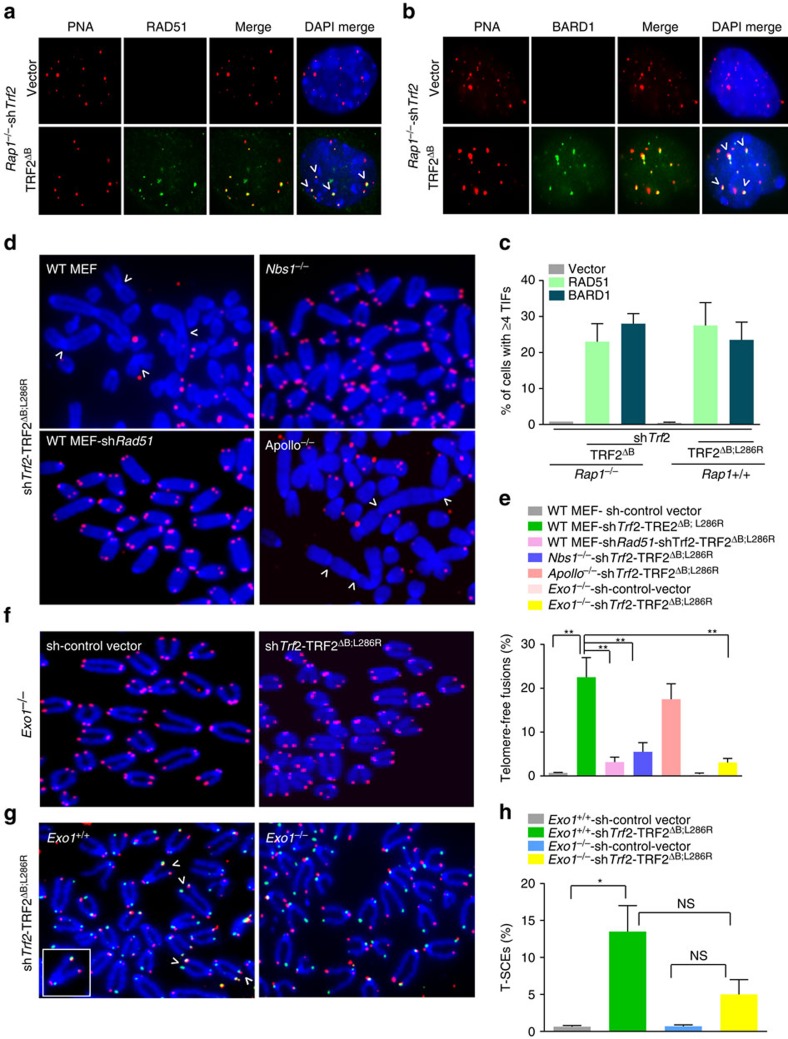
RAP1 and TRF2^B^ repress homologous recombination-mediated repair at telomeres. Telomeric localization of RAD51 (**a**) and BARD1 (**b**) in *Rap*1^*−/−*^ MEFs reconstituted with TRF2^ΔB^, followed by removal of endogenous TRF2 by sh*Trf2.* Telomeres were visualized with PNA-FISH (red), antibodies were used to visualize the presence of RAD51 or BARD1 on telomeres (green), and DAPI to stain nuclei (blue). (**c**) Quantification of percent of cells with ≥4 RAD51- or BARD1-positive TIFs in *Rap*1^*−/−*^ MEFs expressing vector or TRF2^ΔB^ as shown in **a**,**b** as well as quantification of the percent of *Rap*1^*+/+*^ MEFs expressing vector or TRF2^ΔB; L286R^ with ≥4 RAD51- or BARD1-positive TIFs. Data are the mean of three independent experiments ±s.d.; a minimum of 100 nuclei were examined per experiment. (**d**) *Nbs1*^*−/−*^ and *Apollo*^*−/−*^ MEFs and *Rap1*^*+/+*^ MEFs treated with shRNA against *Rad51* were reconstituted with TRF2^ΔB; L286R^, followed by removal of endogenous TRF2 with sh*Trf2*. Metaphase spreads were prepared and telomere fusions visualized by telomere PNA-FISH (red) and DAPI staining (blue). Arrows point to some fused chromosomes. (**e**) Quantification of telomere-free fusions from representative images shown in **d**. Data represent the average of three independent experiments±s.d.; a minimum of 60 metaphases were examined per experiment (**P<0.001; one-way analysis of variance (ANOVA)). (**f**) Metaphase spreads were prepared from *Exo*1^*−/−*^ MEFs expressing vector or TRF2^ΔB; L286R^ and telomere fusions were visualized by telomere PNA-FISH (red) and DAPI staining (blue). (**g**) Telomere sister chromatid exchanges (T-SCEs) were visualized in metaphase spreads from *Exo*1^*+/+*^ and *Exo*1^*−/−*^MEFs expressing vector or TRF2^ΔB; L286R^ by chromosome orientation (CO)-FISH. FITC-OO-(TTAGGG)_4_ (green) was used to visualize the leading strand and Tam-OO-(CCCTAA)_4_ (red) was used to visualize the lagging strand. Arrows point to some T-SCEs. (**h**) Quantification of T-SCEs from representative image shown in **g**. Data are representative of the mean from two independent experiments±s.d.; a minimum of 40 metaphases were analysed per experiment (*P<0.01; one-way ANOVA). NS, not significant.

Given our previous observation that RAP1 is required to repress HDR at mouse telomeres, we postulated that rapid loss of telomeric DNA observed in the absence of RAP1 and TRF2^B^ could be due to aberrant HDR at telomeres. A key step in HDR is the initial 5′–3′ resection of double-stranded DNA to generate 3′ ssDNA by the MRE11–RAD50–NBS1 (MRN complex) and CTIP for loading of recombination proteins (including RAD51) to initiate homologous pairing and strand exchange^27^,^28^. At telomeres, t-loops form when the 3′ ss G-overhang invades into the duplex region of telomeres^29^. The ss G-overhang was significantly elongated in WT MEFs expressing TRF2^ΔB; L286R^ (Fig. 1f and Supplementary Fig. 1k, 1l), suggesting that it could favour strand invasion by HR and the formation of t-loops. In support of this notion, we found that both RAD51 and BARD1, a component of the BRCA1 breast cancer susceptibility HDR protein complex, readily accumulate at telomeres in *Rap1*^*−/−*^ MEFs expressing TRF2^ΔB^ and in WT MEFs expressing TRF2^ΔB; L286R^ (Fig. 3a–c and Supplementary Fig. 3a). We next expressed TRF2^ΔB; L286R^ in either sh*Rad51* depleted or *Nbs1*^*−/−*^ MEFs^30^ and analysed metaphase spreads. Strikingly, removal of *Rad51* or *Nbs1* reduced the formation of telomere-free fusions by six to tenfold, respectively, revealing the importance of HR proteins in the fusion process (Fig. 3d,e and Supplementary Fig. 3b). We also tested the requirement for the Apollo/SNM1B nuclease in generating telomere-free fusions. Apollo/SNM1B is a 5′ to 3′ exonuclease recruited to telomeres by TRF2 and is responsible for the generation of the 3′ overhang in newly replicated leading-strand telomeres^31^,^32^,^33^. Telomere-free fusions were abundant in *Apollo*^*−/−*^ MEFs expressing TRF2^ΔB; L286R^ (Fig. 3d,e), suggesting that the Apollo/SNM1B nuclease is not required for the generation of telomere-free fusions. We next turned to the 5′ to 3′ exonuclease EXO1, since recent studies revealed that it plays an important role in HDR, likely in the lengthening of the ssDNA substrate necessary for HR^34^,^35^,^36^. To address the contribution of EXO1 in HR-mediated telomere recombination, we expressed TRF2^ΔB; L286R^ in *Exo1*^+/+^ *and Exo1*^*−/−*^ MEFs. Similar to the phenotype observed in *Rad51*- or *Nbs1*-depleted cells, telomere-free fusions were reduced by sevenfold in *Exo1*^*−/−*^ MEFs (Fig. 3e,f). These results suggest that EXO1 is required for the elongation of ss G-overhangs to promote the formation of telomere-free fusions. In support of this notion, localization of RAD51 to telomeres was completely abolished in *Exo1*^*−/−*^ MEFs expressed TRF2^ΔB; L286R^ (Supplementary Fig. 3c,d). Finally, chromosome-orientation (CO)-fluorescence *in situ* hybridization (FISH), a measure of HDR between sister telomeres, revealed that T-SCEs were elevated at telomeres in WT, but not in Exo1^*−/−*^ MEF expressing TRF2^ΔB; L286R^ (Fig. 3g,h). Taken together, our data suggest that both the rapid telomere loss and telomere-free fusion phenotypes observed in the absence of functional RAP1 and TRF2^B^ are due to increased HDR at telomeres.

### TRF2^B^-RAP1 repress PARP1 localization to telomeres

**Figure 4:**
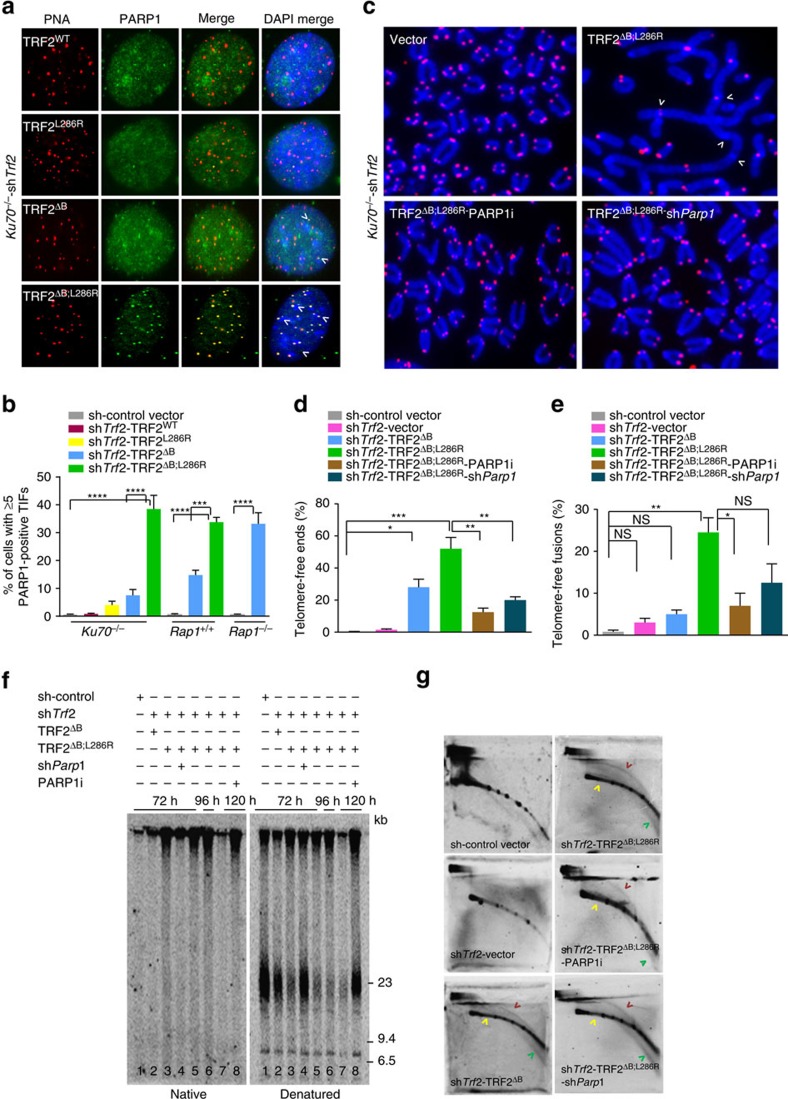
PARP1 promotes rapid telomere attrition and telomere-free fusions in the absence of RAP1 and TRF2^B^. (**a**) Localization of PARP1 in *Ku70*^*−/−*^ MEFs expressing the indicated DNA constructs, followed by removal of endogenous TRF2 with sh*Trf2*. Telomeres were visualized with telomere PNA-FISH (red), anti-PARP1 antibody to visualize PARP1 (green) and DAPI staining to visualize nuclei (blue). (**b**) Quantification of percent of cells with ≥5 PARP1-positive TIFs from representative images shown in **a** as well as in *Rap1*^*+/+*^ or *Rap1*^*−/−*^ MEFs expressing the indicated DNA constructs, followed by removal of endogenous TRF2 with sh*Trf2*. *Ku70*^*−/−*^, *Rap1*^+/+^ and *Rap1*^*−/−*^ MEFs expressing shRNA against control vector do not display any PARP1 foci. Data are averaged from two independent experiments ±s.d.; a minimum of 175 nuclei were scored per experiment. ***P<0.001, ****P<0.0001; one-way analysis of variance (ANOVA). (**c**) Metaphase spreads were prepared from *Ku70*^*−/−*^ MEFs expressing the indicated DNA constructs, followed by removal of endogenous TRF2 with sh*Trf2*. Some cells were also treated with 0.5 μM PARP1 inhibitor (PARP1i) or sh*Parp1* to deplete endogenous PARP1. Telomere fusions were visualized by telomere PNA-FISH (red) and DAPI (blue). Arrows point to several fused chromosomes. (**d**) Quantification of telomere-free chromosome ends as shown in **c**. Data represent the average of two independent experiments ±s.d.; a minimum of 60 metaphases were examined per experiment (*P<0.01, **P<0.001, ***P<0.0001; one-way ANOVA). (**e**) Quantification of telomere-free fusions in **c**. Data represent the average of two independent experiments ±s.d.; a minimum of 60 metaphases were examined per experiment (*P<0.01, **P<0.001; one-way ANOVA). NS, not significant. (**f**) *Ku70*^*−/−*^ MEFs expressing the indicated DNA constructs followed by removal of endogenous TRF2 with sh*Trf2*. Some cells were also treated with sh*Parp1* or with 0.5 μM of the PARP1, PJ34 inhibitor. Time points reflect number of hours after addition of sh*Trf2*: lanes 1–5: 72 h; lane 6: 96 h; lanes 7–8: 120 h. Genomic DNA was fractionated with a clamped homogenous electric field (CHEF) gel, then in-gel hybridization was performed using radiolabelled (CCCTAA)_4_ probes to detect the 3′ single-stranded overhang under native conditions (left) and under denaturing conditions to detect total TTAGGG repeats (right). Molecular weights are displayed on the right. (**g**) Genomic DNA isolated from *Ku70*^*−/−*^ MEFs expressing the indicated DNA constructs and/or the PARP1, PJ34 inhibitor, followed by removal of endogenous TRF2 with sh*Trf2*, were fractionated by neutral two-dimensional (2D) gel electrophoresis. The gel was denatured, dried and hybridized *in situ* with a radiolabelled (CCCTAA)_4_ oligonucleotide probe to detect single-stranded telomere DNA (green arrow), double-stranded telomere DNA (yellow arrow) and telomeric (T) circles (red arrow).

Poly(ADP-ribose) polymerase 1 (PARP1), a protein that plays multiple roles in DNA damage repair, interacts with TRF2 and is thought to play a role in the repair of critically shortened telomeres^37^,^38^. Compared with MEFs expressing WT TRF2, a 5-fold and 15-fold increase in PARP1 localization to telomeres was observed in *Ku70*^*−/−*^ and WT MEFs expressing TRF2^ΔB^, respectively (Fig. 4a,b). This result suggests that the TRF2 basic domain plays an important role in repressing PARP1 recruitment to telomeres, and that Ku70 facilitates this repression. Interestingly, the number of PARP1 foci increased ∼37-fold in both WT and *Ku70*^*−/−*^ MEFs expressing TRF2^ΔB; L286R^, suggesting that complete repression of PARP1 localization to telomeres requires both RAP1 and TRF2^B^. In support of this notion, compared with *Rap1*^*−/−*^ MEFs expressing vector control, PARP1 foci increased ∼35-fold in *Rap1*^*−/−*^ MEFs expressing TRF2^ΔB^ (Fig. 4b). To ascertain whether PARP1 plays a role in the formation of telomere-free fusions, we treated *Ku70*^*−/−*^ MEFs expressing TRF2^ΔB; L286R^ with either the PARP inhibitor PJ34^39^ or a shRNA against *Parp1* (Supplementary Fig. 4a). Depletion of *Parp1* significantly reduced the number of telomere signal-free ends, telomere-free fusions and global telomere loss as assayed by TRF Southern blot analysis (Fig. 4c–f and Supplementary Fig. 4b,c). We also examined the presence of extrachromosomal telomeric circles (TC) in MEFs expressing TRF2^ΔB; L286R^. T-circles are the products of t-loop HR, aberrant HR-mediated resolution of telomeric t-loops and stems from the deletion of large segments of telomeric DNA, leading stochastic telomere shortening^20^. Although t-loop HR was prominent in *Ku70*^*−/−*^ MEFs expressing TRF2^ΔB; L286R^, we found that PARP1 inhibition reduced the number of TCs in these cells (Fig. 4g and Supplementary Fig. 4d,e). Finally, we used an *in vitro* TC amplification assay^40^ to confirm the presence of TCs in cells lacking both RAP1 and TRF2^B^. We found a high level of Phi29-dependent TCs in WT MEFs expressing TRF2^ΔB; L286R^ and a corresponding decrease in total telomere length by TRF Southern blot analysis (Supplementary Fig. 4f–h). Taken together, our data reveal that both RAP1 and TRF2^B^ are required to fully repress PARP1 localization to telomeres to prevent the initiation of t-loop HR and rapid telomere loss due to excision of telomeres as a circle.

### TRF2^B^-RAP1 repress SLX4 localization and t-loop resolution

**Figure 5:**
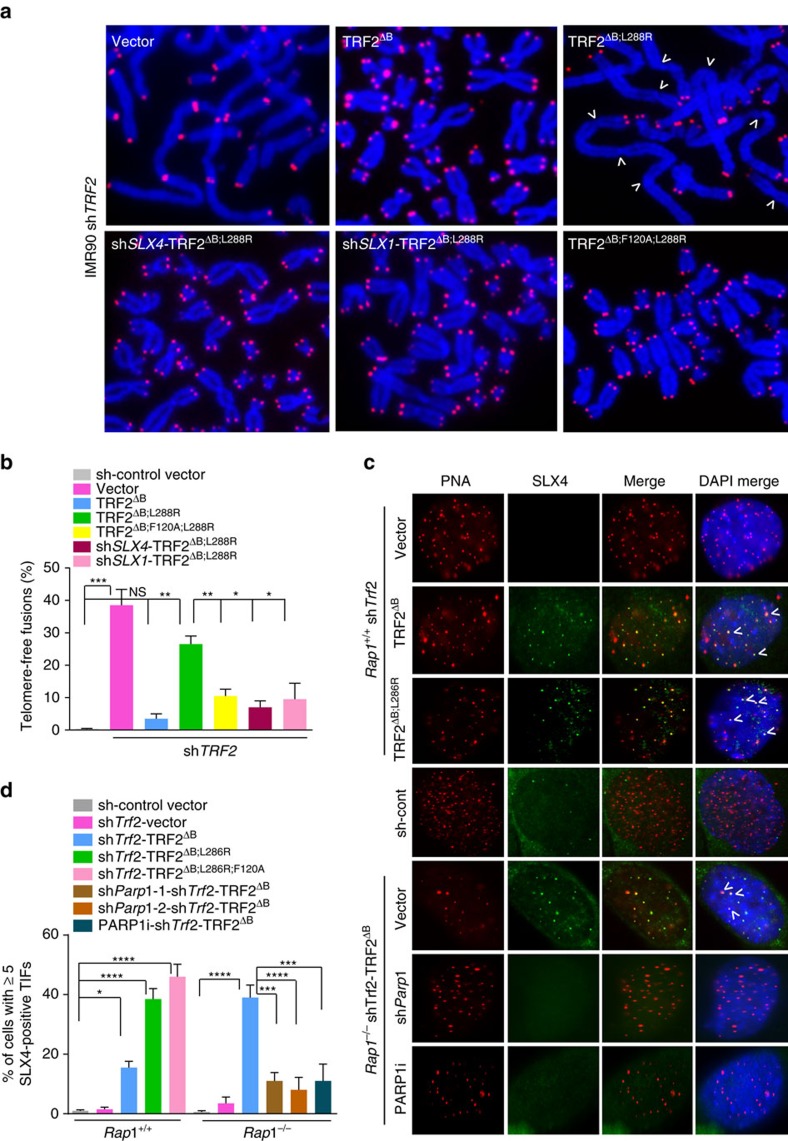
TRF2^B^ and RAP1 repress SLX4 localization to telomeres and the generation of telomere-free chromosome fusions. (**a**) Metaphase spreads were prepared from IMR90 cells expressing the indicated DNAs, followed by removal of endogenous TRF2 with sh*Trf2*. Telomeres visualized by telomere PNA-FISH (red) and chromosomes by DAPI (blue). Arrows point to several chromosome fusion sites. (**b**) Quantification of telomere-free fusions from representative images shown in **a**. Data are averaged from two independent experiments ±s.d.; a minimum of 40 metaphases were examined per experiment (*P<0.01, **P<0.001, ***P<0.0001; one-way analysis of variance (ANOVA)). (**c**) Telomere localization of endogenous SLX4 in *Rap1*^*+/+*^ and *Rap1*^*−/−*^ MEFs expressing the indicated DNA constructs, some treated with either *Parp1* shRNA or 0.5 μM PARP1 inhibitor, followed by removal of endogenous TRF2 with sh*Trf2*. Telomeres were visualized with telomere PNA-FISH (red), anti-SLX4 antibody (green) and DAPI (blue). (**d**) Quantification of percent of cells with≥5 SLX4-positive TIFs from representative images shown in **c**. Data are averaged from two independent experiments±s.d.; a minimum of 200 cells scored per experiment. (*P<0.01, ***P<0.001; ****P<0.0001; one-way ANOVA).

The SLX4 resolvase is a scaffold protein that assembles the endonucleases SLX1, XPF-ERCC1 and MUS81 to sites of DNA damage and dysfunctional telomeres^41^,^42^,^43^,^44^,^45^,^46^,^47^. We have previously shown that human TRF2 recruits SLX4 to telomeres via its TRFH domain, leading to telomere shortening and t-loop resolution^45^. Interestingly, although mouse SLX4 lacks a TRFH-binding motif, it is nevertheless required for telomere maintenance^46^. To determine whether SLX4 plays a role in the generation of telomere-free fusions, we expressed TRF2^ΔB; L288R^ in sh*SLX4*-treated IMR90 cells and then deplete endogenous TRF2 with sh*TRF2*. Although ∼28% of all chromosome ends in cells expressing TRF2^ΔB; L288R^ displayed telomere-free fusions, shRNA-mediated depletion of *SLX4* reduced these fusions ∼3-fold, suggesting that SLX4 is required to generate telomere-free fusions (Fig. 5a,b and Supplementary Fig. 5a). To test whether SLX4 recruitment to telomeres by TRF2 requires a functional TRF2^TRFH^ domain, we mutated phenylalanine 120 into an alanine^45^ to generate TRF2^ΔB; F120A; L288R^. Compared with the number of telomere-free fusions observed in IMR90 cells expressing TRF2^ΔB; L288R^, reconstitution with TRF2^ΔB; F120A; L288R^ also reduced telomere-free fusions ∼3-fold, to levels similar to those observed in sh*SLX4*-treated cells (Fig. 5a,b). Further supporting the notion that SLX4 recruitment to telomeres is required for telomere attrition, expression of SLX4 in *Rap1*^*−/−*^ MEFs increased the number of chromosome ends lacking telomeric signals (Supplementary Fig. 5b,c). Finally, we show that depletion of the endonuclease *SLX1* in IMR90 cells reduced the number of telomere-free fusions threefold (Fig. 5a,b). These results support the notion that human RAP1 and TRF2^B^ cooperate to repress SLX4 localization to telomeres that would otherwise result in catastrophic telomere attrition and telomere-free fusions.

We next examined the localization of endogenous mouse SLX4 in both WT and *Rap1*^*−/−*^ MEFs. Mouse SLX4 cannot interact with the TRF2^TRFH^ domain^45^,^46^, and consequently the TRF2^F120A^ mutation was unable to abrogate localization of SLX4 to telomeres, or prevent the formation of t-circles, global telomere deletion and telomere-free fusions (Fig. 5d and Supplementary Fig. 4f–h). We were also unable to detect endogenous SLX4 at telomeres in WT MEFs expressing vector control (Fig. 5c,d). However, ≥5 SLX4-positive TIFs were detected in 14% of WT MEFs expressing TRF2^ΔB^ (Fig. 5c,d), supporting a role for TRF2^B^ in repressing murine SLX4 localization to telomeres. We found that 38% of WT MEFs expressing TRF2^ΔB; L286R^ displayed ≥5 SLX4-positive TIFs, reinforcing the view that both RAP1 and TRF2^B^ cooperate to repress mouse SLX4 localization to telomeres. Similar results were observed in *Rap1*^*−/−*^ MEFs expressing TRF2^B^ (Fig. 5c,d).

Finally, we found that murine SLX4 localization to telomeres is dependent upon functional PARP1, as depletion of *Parp1* by either shRNA expression or treatment with the PARP1 PJ34 inhibitor in *Rap1*^*−/−*^ MEFs expressing TRF2^ΔB^ resulted in ∼3.5- and 5-fold reduction in SLX4 localization to telomeres, respectively (Fig. 5c,d). Taken together, our data strongly support the notion that RAP1 cooperates with TRF2^B^ to repress PARP1 localization to telomeres, which in turn prevents SLX4's telomeric localization.

### RAP1^BRCT^ and RAP1^Myb^ prevent telomere deletions and fusions

**Figure 6:**
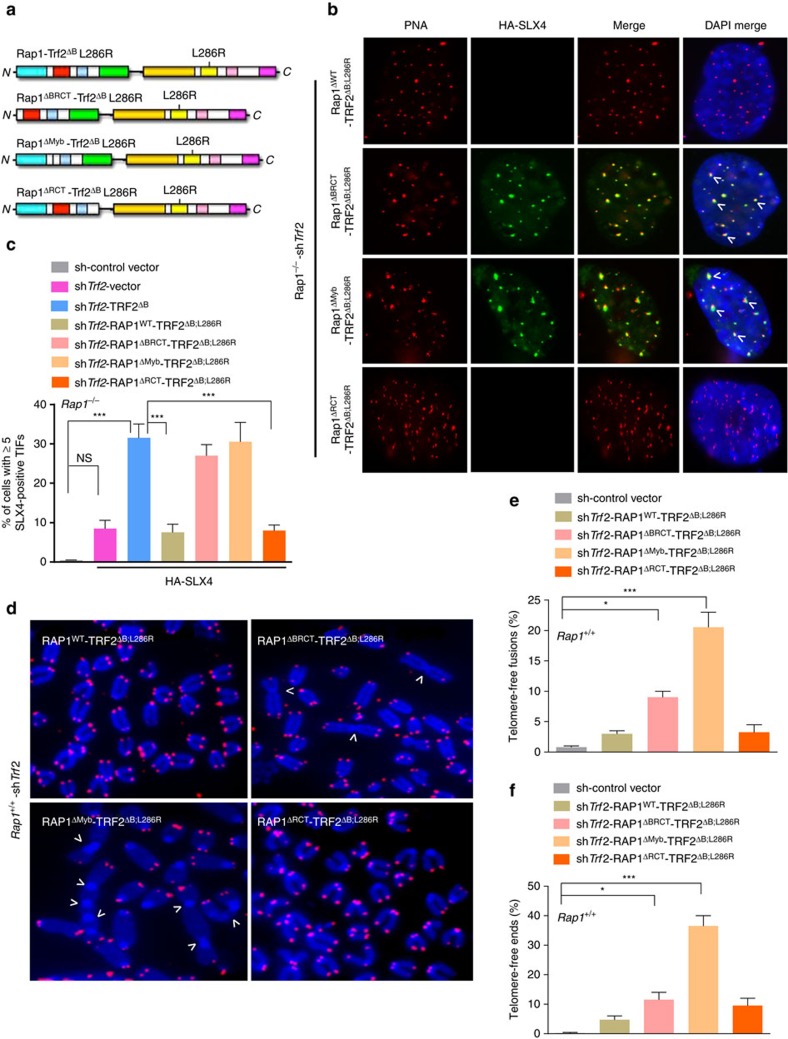
The RAP1 Myb domain is required to repress telomere attrition and chromosome fusions. (**a**) Schematic of RAP1-TRF2^ΔB; L286R^ fusion proteins used. RAP1 domains are coloured, with cyan, red, light blue and green denote, respectively, the BRCT, Myb, coiled–coil and RCT domains. (**b**) Telomeric localization of HA-SLX4 in *Rap1*^*−/−*^ MEFs expressing indicated DNAs, followed by removal of endogenous TRF2 with sh*Trf2*. Telomeres were visualized by telomere PNA-FISH (red), HA-SLX4 by anti-HA antibody (green) and chromosomes by DAPI (blue). (**c**) Quantification of percent of cells with ≥5 HA-SLX4-positive TIFs shown in **b**. Data are averaged from two independent experiments ±s.d.; a minimum of 200 cells scored per experiment. (***P<0.0001, one-way analysis of variance (ANOVA)). NS, non-significant values. (**d**) *Rap1*^*+/+*^ MEFs were reconstituted with the indicated DNA constructs, followed by removal of endogenous TRF2 with sh*Trf2*. Metaphases were prepared and telomere fusions visualized by telomere PNA-FISH (red) and DAPI (blue). Arrows point to chromosome fusion sites. (**e**) Quantification of telomere-free fusions from representative images shown in **d**. Data are averaged from two independent experiments ±s.d.; a minimum of 50 metaphases were examined per experiment (*P<0.01, ***P<0.001; one-way ANOVA). (**f**) Quantification of telomere-free ends from representative images shown in **d**. Data are averaged from two independent experiments ±s.d.; 50 metaphases were scored per experiment (*P<0.01, ***P<0.0003; one-way ANOVA).

Our data suggest that WT RAP1 and TRF2^B^ are both required to prevent SLX4 from initiating aberrant HDR at telomeres. To dissect which RAP1 domain(s) are able to cooperate with TRF2^B^ to repress SLX4 localization to telomeres, we generated a RAP1^WT^-TRF2^ΔB; L286R^ fusion construct as well as various RAP1 domain mutants, in which either the RAP1 BRCT, Myb or the C-terminal (RCT) domains were deleted (Fig. 6a). All RAP1-TRF2 constructs localized to telomeres and were able to repress CHK2 phosphorylation after removal of endogenous TRF2 (Supplementary Fig. 6a,b). Previous reports suggest that both the RAP1 BRCT and Myb domains are important for telomere length regulation^37^,^48^. Using a HA-SLX4 construct to better visualize SLX4 foci at telomeres, we observed ≥5 HA-SLX4-positive TIFs in ∼8.5% of sh*Trf2*-depleted *Rap1*^*−/−*^ MEFs, a level of TIF formation similar to what we saw with endogenous SLX4 under comparable experimental conditions (Figs 5d and 6b,c). We found that 34% of *Rap1*^*−/−*^ MEFs expressing TRF2^B^ displayed ≥5 HA-SLX4-positive TIFs, and this high level of TIF formation was not reduced when either RAP1^ΔMyb^-TRF2^ΔB; L286R^ or RAP1^ΔBRCT^-TRF2^ΔB; L286R^ were expressed in *Rap1*^*−/−*^ MEFs (Fig. 6b,c). In contrast, expression of RAP1^WT^-TRF2^ΔB; L286R^ or the RAP1^ΔRCT^-TRF2^ΔB; L286R^ constructs in *Rap1*^*−/−*^ MEFs reduced the number of cells displaying ≥5 HA-SLX4 TIFs to ∼8%. These results suggest that both the RAP1^Myb^ and RAP1^BRCT^ domains are required to cooperate with TRF2^B^ to repress SLX4 localization to dysfunctional telomeres.

Examination of metaphase spreads revealed that while both the RAP1^Myb^ and RAP1^BRCT^ domains are also required to repress the formation of both telomere-free fusions and signal-free ends, the RAP1^Myb^ domain appears to be crucial for these processes, as telomere-free fusions and signal-free ends were observed involving ∼22% and ∼35%, respectively, of all chromosome ends in WT MEFs expressing RAP1^ΔMyb^-TRF2^ΔB; L286R^ (Fig. 6d–f). Together, these results suggest that the RAP1^MYB^ domain is essential to cooperates with TRF2^B^ to repress both t-loop HR and formation of telomere-free fusions.

## Discussion

The mammalian TRF2-RAP1 heterodimer possesses important telomere end-protective functions essential to maintain genome stability. TRF2 is the main component of the Shelterin complex that represses C-NHEJ-mediated chromosome fusions, and it also participates in the repression of HDR^6^,^15^,^19^. RAP1 also functions to block HDR at mouse telomeres by preventing T-SCEs, however, it is puzzling why this function is not observed in human cells^49^. As RAP1 is the most highly conserved Shelterin component and also plays a role in transcription^16^,^49^, an important question is whether this evolutionary conservation is due to RAP1's roles in telomere end protection or transcriptional regulation. Here we present strong evidence that mammalian RAP1 is essential to protect telomere from HR-mediated repair. RAP1 cooperates with TRF2 via its basic domain to repress PARP1 localization to telomeres, and this in turn prevents SLX4's telomeric localization. In the absence of RAP1 and TRF2^B^, PARP1, SLX4 and proteins involved in HDR localize to telomeres to promote rapid telomere resection, resulting in catastrophic telomere loss, formation of telomere-free chromosome ends and massive telomere-free chromosome fusions in both mouse and human cells. Our results highlight the importance of the RAP1-TRF2 heterodimer in protecting telomeres from inappropriate processing by the HR pathway.

Mammalian telomeres can adopt a t-loop structure, in which the 3′ ss overhang is sequestered into homologous sequences through strand-invasion^29^,^50^. This structure is thought to shield the 3′ telomeric terminus from DNA damage sensors including the MRN complex, thereby preventing inappropriate activation of a DNA damage response. T-loop formation requires TRF2, which through topological changes in bound DNA stimulates strand invasion^51^,^52^. The TRF2^B^ domain is required to stabilize this strand invasion by binding to the resulting HJ^52^,^53^,^54^. Although t-loop formation is thought to be important for telomere end protection, t-loops also resemble HR intermediates and if not appropriately protected, are targets for recombination endonucleases able to process HJs. Inappropriate processing of t-loops by HJ resolvases, including the SLX4 resolvase complex, leads to telomere resection, t-circle formation and telomere shortening^20^,^40^,^55^. These aberrant telomeric products are found in cells expressing TRF2^ΔB^, thus implicating the TRF2^B^ domain in preventing t-loop formation and telomere shortening. In agreement with previously published results^20^,^21^,^40^, our data reveal that TRF2^B^ limits SLX4-SLX1 resolvase activities at telomeres by preventing its localization to telomeric DNA. This is likely due to TRF2^B^'s ability to represses PARP1 localization to telomeres, and we show that SLX4 localization to telomeres requires functional PARP1. As PARP1 participates in a slew of repair reactions at damaged DNA, including HDR, limiting its access to telomeres would prevent telomere HDR. Indeed, shRNAs against *PARP1*, or the use of a PARP1 inhibitor, dramatically reduced the formation of t-circles. We speculate that localization of human SLX4 to telomeres requires both interaction with TRF2's TRFH domain^45^ as well as modifications by PARP1. In support of this notion, a recent report revealed that SLX4 interacts with PARP1, and that PARylation is required to recruit SLX4 to sites of damaged DNA^56^.

**Figure 7:**
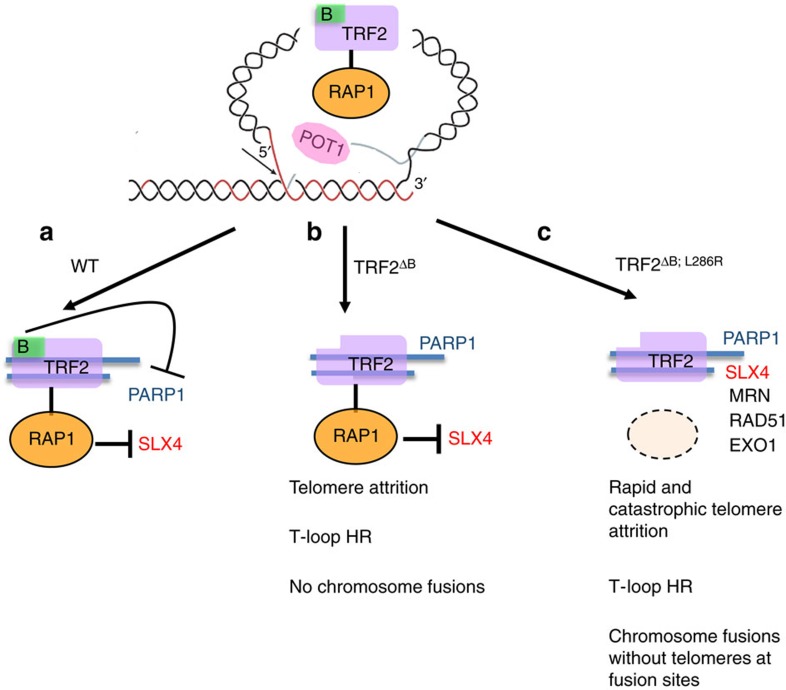
Schematic model of the roles of TRF2 and RAP1 in repressing aberrant HR at telomeres. (**a**) In WT cells, TRF2 and RAP1 form a stable complex and cooperate to protect chromosome ends from inappropriate repair. RAP1 prevents SLX4 localization to telomeres, whereas the basic domain of TRF2 blocks PARP1 localization to telomeres. In addition, TRF2 also functions as a docking site to recruit other DNA processing and repair factors to telomeres to promote normal telomere metabolism. (**b**) In the absence of TRF2^B^, PARP1 localizes to telomeres, increasing telomere t-circle formation and telomere attrition without generating end-to-end chromosome fusions. (**c**) Removal of both RAP1 and TRF2^B^ results in the localization of both SLX4 and PARP1 to telomeres, promoting the recruitment of factors involved in HR, including RAD51, EXOI and the MRN complex to telomeres. These factors help promote catastrophic telomere deletion via t-loop HR and the formation of telomere-free fusions.

Given the importance of the TRF2 basic domain in telomere end protection, it was puzzling why cells expressing TRF2^ΔB^ still retained the majority of telomeres and did not exhibit increased end-to-end chromosome fusions^20^. The data presented here reveal that this is due to RAP1's protective functions at telomeres. Catastrophic telomere loss and telomere-free fusions are only detected in cells lacking both RAP1 and TRF2^B^. Telomere-free fusions do not contain any detectable telomeric signals at fusion sites and appear completely distinct from the chromosome fusions observed when TRF2 is removed from telomeres, in which robust telomeric signals are almost always present at fusion sites^23^,^24^. As telomeres possess 5′ to 3′ polarity, joining chromosome ends that contain telomeric sequences could only occur through C-NHEJ- or A-NHEJ-mediated DNA repair^24^,^25^,^26^. We show that telomere-free fusions still occur when C-NHEJ- and A-NHEJ-mediated DNA repair pathways are abrogated. This type of chromosome fusion requires proteins involved in HR, including NBS1, RAD51, EXO1 and PARP1, revealing that telomere-free fusions occur by HDR. Although reconstitution of TRF2^ΔB^ is able to protect telomeres devoid of WT TRF2 from activating a DNA damage response (DDR) and C-NHEJ-mediated repair, in the absence of RAP1, it is unable to prevent rapid telomere attrition and formation of telomere-free fusions. We found that HDR-mediated telomere-free fusions require extensive EXO1-mediated telomere end resection, generating elongated G-overhangs, which likely favours HR-mediated strand invasion. Our results therefore suggest that the RAP1-TRF2 heterodimer might also play a role in regulating telomere end resection, perhaps by recruiting the POT1-TPP1 complex to block EXO1 access to the 5′ C-strand. Failure to do so would generate a long 3′ overhang favourable for HR-mediated strand invasion into the distal double-stranded region of telomeric DNA. This would result in branch migration and resolution of the HJ by the SLX4 resolvase to form t-circles and linear excised telomeric ends that can undergo repeated recombination cycles, leading to catastrophic telomere loss. Subsequent fusion of the chromosomal ends lacking telomeric sequences would generate fusion sites without telomere DNA (Fig. 7a–c).

*S. pombe* and *S. cerevisiae* reset hyper elongated telomeres to WT lengths via a t-loop HR-mediated mechanism termed telomere rapid deletion (TRD)^57^,^58^. Given that t-loops are found at the ends of chromosomes in many diverse organisms^59^,^60^, TRD likely represents an evolutionarily conserved telomere length maintenance mechanism. Our results reveal that the telomere attrition phenotypes associated with deletion of RAP1 and the TRF2 basic domain shares several features with other systems that exhibit telomere deletion, including yeast TRD, *K. lactis* that lacks functional *Rap1* (ref. 61) and human cells lacking *Ku86* (ref. 62). These include the requirement for HR factors and rapid telomere shortening through t-loop formation. However, massive formation of telomere-free chromosome fusions was never observed in any of these other systems and likely represents an extreme consequence of what could happen to chromosome ends if t-loop HR is not properly regulated. Our data suggest that in mammalian cells, RAP1 functions to modulate TRF2's propensity to form t-loops. In the absence of RAP1, TRF2^ΔB^ unleashes aberrant t-loop HR with catastrophic consequences. In support of this hypothesis, RAP1 has been recently shown biophysically to influence the binding of TRF2^B^ to DNA, resulting in improved TRF2 selectivity to single/double-strand junctions of telomeric DNA^63^. It is tempting to speculate that the RAP1^Myb^ domain (and to a lesser extent the RAP1^BRCT^ domain) modulates TRF2^B^'s function in t-loop HR and the formation of telomere-free fusions by limiting the accumulation of PARP1/SLX4 at telomeres. As RAP1 is an adapter protein^64^, it is possible that the RAP1^Myb^ and RAP1^BRCT^ domains directly interact with these proteins to modulate telomeric access, or recruit additional protein(s) to perform this function.

## Methods

### Antibodies and western blot analysis

The antibodies used for western blot analysis were as follows: phospho-CHK1 (Cell Signaling Technology, catalogue no 13,303, dilution 1:1,000), phospho-CHK2 (BD Biosciences, catalogue no. 611,570, dilution 1:2,000), anti γ-H2AX (Millipore, catalogue no. 05–636, dilution 1:2,000). Anti-PARP1 (catalogue no. sc-53,643, dilution 1:1,000), anti-SLX4 (H-39, catalogue no. sc-135,225, dilution 1:1,000), anti-RAD51 (H-92, catalogue no. sc-8,349, dilution 1:500), anti-BARD1 (H-300, catalogue no. sc-11,438, dilution 1:1,000), anti-RAP1 (H-300, catalogue no. sc-28,197, dilution 1:500) and anti-Ligase 3 (6G9, catalogue no. sc- 56,089, dilution 1:500) were from Santa Cruz. Anti-Flag (catalogue no. F3165, dilution 1:2,000), anti-HA (catalogue no. H3663, dilution 1:2,000) and anti-γ-tubulin (clone GTU-488, catalogue no. T6557, dilution 1:5,000) were purchased from Sigma-Aldrich. Anti-mouse TRF2 (dilution 1:1,000) was a kind gift from Dr Jan Karlseder, Salk Institute. For immunoblotting, trypsinized cells were lysed in urea lysis buffer (8 M urea, 50 mM Tris-HCl, pH 7.4, and 150 mM β-mercaptoethanol). The lysate was resolved on SDS–PAGE gel. The separated proteins were then blotted on a nitrocellulose plus membrane (Amersham), blocked with blocking solution (5% non-fat dry milk in PBS/0.1% Tween-20) for at least 1 h and incubated with appropriate primary antibody in blocking solution at least 2 h at room temperature or overnight at 4 °C. The membranes were washed 3 × 5 min with PBS/0.1% Tween-20 and incubated with appropriate secondary antibody in blocking solution for 1 h at room temperature. Chemiluminescence detection was performed using an ECL Western Blotting Detection kit from GE Healthcare.

### Expression vectors and shRNAs

TRF2^WT^, TRF2^L286R^, TRF2^ΔB^ and TRF2^ΔB; L286R^ were cloned into pQCXIP-puro retroviral expression vectors. All the constructs were confirmed by sequencing. Point mutations were introduced using side-directed mutagenesis according to the manufacturer's protocol (Stratagene). *Trf2* shRNA was used to deplete endogenous *Trf2* (ref. 22). Human *TRF2* shRNA in pLKO.1 vector was obtained from Sigma and corresponds to the TRCN0000280026 clone. shRNA against *Rad51*, *Parp1* and *Ligase 3* were gifts from Dr Madalena Tarsounas, Oxford, UK. shRNA against *SLX4* and *SLX1* was a gift from Dr Yie Liu, NIA.

### Cell lines and retroviral infection in cell lines

IMR90 (Dr. Jan Karlseder, Salk), *Rap1*^*−/−*^, MEFs (Dr. Maria Blasco, Spain) *ATM*^*−/−*^*, Ku70*^*−/−*^*, Ligase 4*^*−/−*^ (Chang lab), *Nbs1*^*−/−*^ (Dr. Kenshi Komatsu, Kyoto), *Apollo/Snm1B*^*−/−*^ (Chang lab) and *Exo1*^*−/−*^ MEFs (Dr. Winfried Edelmanna, Einstein) were cultured in DMEM supplemented with 10% FBS and maintained in 5% CO_2_ at 37 °C. For retroviral infection, DNA constructs were transfected into 293T cells using Fugene 6 and packaged into viral particles. Viral supernatant was collected 48–72 h after transfection, filtered through 0.45-μm pore-size membrane and directly used to infect SV40^LT^-immortalized MEFs. To stably express mutant TRF2 constructs in MEFs, MEFs were first infected with shRNA-resistant TRF2 constructs, and endogenous TRF2 was depleted with shRNA against *Trf2*. After 96–120 h, cells selected with 2 μg ml^−1^ puromycin were harvested for chromosome analysis.

### Immunofluorescence and fluorescent *in situ* hybridization

Cells grown on coverslips were fixed for 10 min in 2% (w/v) sucrose and 2% (v/v) paraformaldehyde at room temperature followed by PBS washes. Coverslips were blocked for 1 h in blocking solution (0.2% (w/v) fish gelatin and 0.5% (w/v) BSA in 1 × PBS). The cells were incubated with primary antibodies for 2 h at room temperature. After PBS washes, coverslips were incubated with the appropriate Alexa Fluor secondary antibody for 1 h followed by washes in 1 × PBS with 0.1% Triton. For immunofluorescence-FISH to detect both proteins and telomeres, the secondary antibody was fixed to the primary antibody by incubating labelled cells in 4% paraformaldehyde for 10 min at room temperature^65^. After three PBS washes, 30 μl of freshly prepared peptide nucleic acid (PNA)-FISH hybridization mix (2% BSA, 100 μg ml^−1^ tRNA, 0.6 × SSC, 100% formamide and 10 ng μl^−1^ 5′-Tam-OO-(CCCTAA)_4_-3′ PNA telomere probe (PANAgene)) was added to the coverslips. The coverslips were then denatured at 85 °C on a hot plate for 3 min and incubated overnight in a humidified chamber, washed with solution I (70% formamide, 0.1% Tween-20, 0.1% BSA, 10 mM mM Tris-HCl, pH 7.5) and then solution II (50 mM Tris-HCl, pH 7.5, 150 mM NaCl, 0.1% BSA, 0.1% Tween-20). DNA was counterstained with 4,6-diamidino-2-phenylindole and digital images were captured with a Nikon Eclipse 800 microscope utilizing an Andore CCD camera.

### Chromosome analysis by Telomere PNA-FISH and CO-FISH

Cells were treated with 0.5 μg ml^−1^ of Colcemid for 4–7 h before chromosome harvest. Metaphase spreads were fixed in 4% formaldehyde in PBS for 10 min and then dehydrated with sequential rinses in 70, 85 and 100% ethanol for 2 min each and air dried. Telomere PNA-FISH was performed with 10 ng μl^−1^ 5′-Tam-OO-(CCCTAA)_4_-3′ probe in PNA-FISH hybridization mix (2% BSA, 100 μg ml^−1^ tRNA, 0.6 × SSC, 100% formamide)^66^,^67^. For CO-FISH, fixed metaphase spreads were incubated sequentially with 10 ng μl^−1^ 5′-Tam-OO-(CCCTAA)_4_-3′ and 10 ng μl^−1^ 5′-FITC-CO-(TTAGGG)_4_-3′ probes in PNA-FISH hybridization mix^24^. All images were captured on a Nikon Eclipse 800 microscope and processed with MetaMorph Premier (Molecular Devices). The percent of chromosome fusions observed is defined as: total number of chromosome fusions in 30–50 metaphase spreads analysed divided by the total number of chromosomes examined × 100%. Telomere-free fusions are defined as end-to-end chromosome fusions without any telomeric signals at sites of fusion.

### Telomere length analysis and G-Strand overhang assays

For in-gel detection of telomere length and G-stand overhang, a total of 1–2 × 10^6^ cells were suspended in PBS, mixed 1:1 with 1.8% agarose in 1 × PBS and cast into plugs. The plugs were then digested overnight at 50 °C with 20 mg ml^−1^ Proteinase K (Roche) in 10 mM sodium phosphate (pH 7.2) and 0.5 mM EDTA and 1% sodium lauryl sarcosine. DNA in plugs were subsequently digested by *Hin*f1/*Rsa*1 overnight at 37 °C. The next morning, plugs were washed once with 1 × TE and equilibrated with 0.5 × TBE. The plugs were loaded onto a 0.8% agarose gel in 0.5 × TBE and run on a CHEF-DRII pulse field electrophoresis apparatus (Bio-Rad). The electrophoresis parameters were as follows: Initial pulse: 0.3 s, final pulse: 16 s, voltage: 6 V cm^−1^, run time: 14 h. Dried gel pre-hybridized with Church mix for 2 h at 55 °C and hybridized overnight at 55 °C in Church mix with ^32^P-labelled T_2_AG_3_ oligonucleotides. After hybridization, the gel was washed three times for 30 min with 4 × SSC/0.1% SDS at 37 °C, thrice with 4 × SSC/0.1% SDS at 55°C and exposed to a phosphoimager screen overnight. After exposure, the screen was scanned on a STORM phosphoimager (Molecular Dynamics) and the gel was subsequently denatured and hybridized using the same probe.

### 2D-TRF Southern assay

Thirty micrograms of total genomic DNA digested with *Hin*f1/*Rsa*1 was resolved in the first dimension in 0.4% agarose gel in 0.5 × TBE at 26 V for 15 h. Ethidium bromide-stained gel slices were rotated 90° from the first gel and cast in a 1% agarose gel and resolved at 126 V for 4 h. Dried gel were denatured and subsequently hybridized as described above.

### Telomere circle assay

T-circle assay was performed with 1.0 μg genomic DNA digested with *Alu*I and *Hin*fI and then annealed with 10 μM Thio-(C_3_TA_2_)_3_. Rolling circle amplification was performed with and without Phi29 DNA polymerase^40^. Amplified product were subjected to Southern blotting and hybridized with ^32^P-labelled T_2_AG_3_ oligonucleotides. Southern blot images were scanned on a STORM phosphoimager (Molecular Dynamics). The level of ^32^P incorporation in the Phi29-negative control samples was subtracted from the samples that contained the Phi29 DNA polymerase.

## Additional information

**How to cite this article:** Rai, R. *et al*. TRF2-RAP1 is required to protect telomeres from engaging in homologous recombination-mediated deletions and fusions. *Nat. Commun.* 7:10881 doi: 10.1038/ncomms10881 (2016).

## Supplementary Material

Supplementary Information Supplementary Figures 1-7
