# Supplementary material for: TRF2-RAP1 is required to protect telomeres from engaging in homologous recombination-mediated deletions and fusions
Source: Nat Commun. 2016 Mar 4;7:10881. doi: 10.1038/ncomms10881 (PMC4785230; doi:10.1038/ncomms10881)
Supplement: Supplementary Information — Supplementary Figures 1-7 [file ncomms10881-s1.pdf]

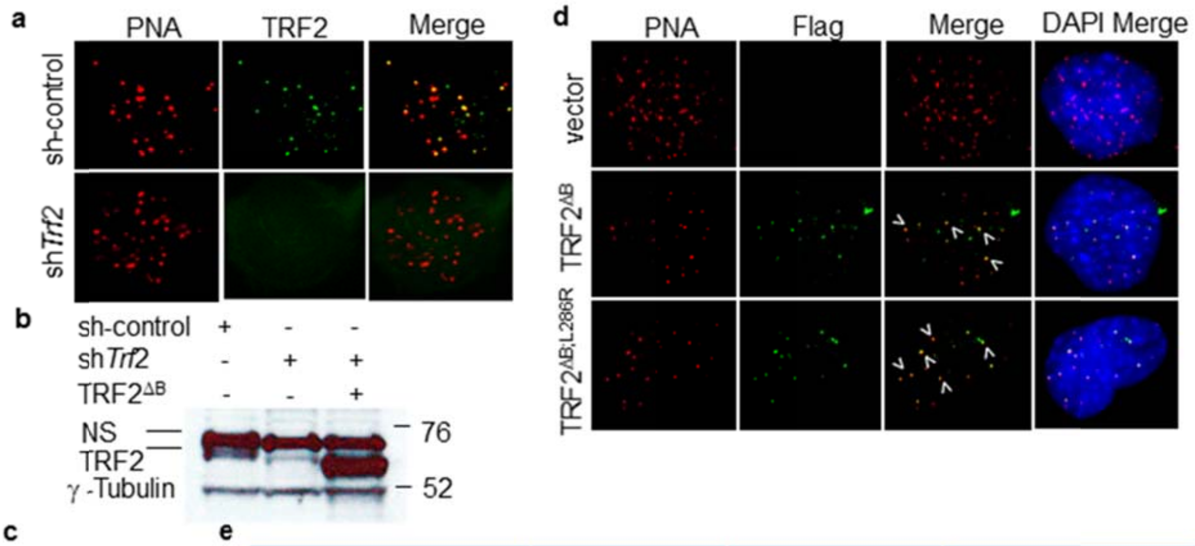

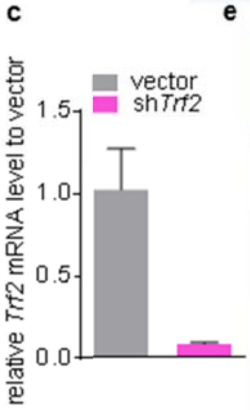

e

Genotype	Total no. of chromosomes scored	Fusions with telomere at the site of fusions	Fusions without telomere at the site of fusions
Wild Type MEF +sh-control vector	3456	3 (0.09%)	0
Wild Type MEF +sh*Trf2*+vector	5124	2663 (52%)	113 (2.2%)
Wild Type MEF +sh*Trf2*+TRF2$^{\Delta B}$	5120	230 (4.5%)	95 (1.9%)
Wild Type MEF +sh*Trf2*+TRF2$^{\Delta B;L286R}$	7214	288 (4%)	2308 (32%)

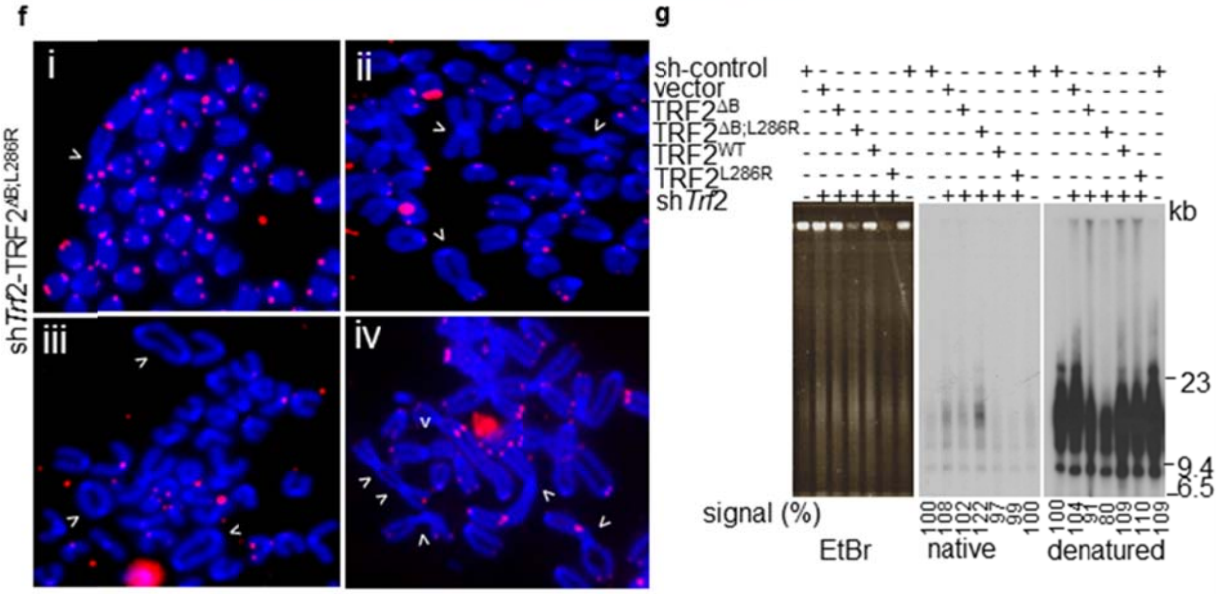

Supplementary Figure 1 (**a**) Immunofluorescence analysis to monitor the depletion of TRF2. Fixed cells were stained with anti-TRF2 antibody (green), telomeres visualized by PNA-FISH (red), and DAPI to stain nuclei (blue). (**b**) Immunoblots to detect $TRF2^{\Delta B}$ in WT MEFs depleted of endogenous TRF2 and reconstituted with $TRF2^{\Delta B}$. γ-Tubulin used as loading control. NS denotes nonspecific band above TRF2 band. Endogenous TRF2 was not detected with this anti-TRF2 antibody. (**c**) *Trf2* mRNA levels detected by real time RT-PCR in WT MEFs treated with sh-control vector or sh*Trf2*. (**d**) Telomeric localization of retrovirally expressed Flag-$TRF2^{\Delta B}$ and Flag-$TRF2^{\Delta B;\ L286R}$ in WT MEFs. Telomeres were visualized with PNA-FISH (red), anti-Flag antibody to visualize TRF2 mutants (green), and DAPI for nuclei (blue). (**e**) Quantification of the number of chromosome fusions with or without telomeric signals at fusion sites in WT MEFs treated with vector, sh*Trf2*, sh*Trf2* and $TRF2^{\Delta B}$ or sh*Trf2* and $TRF2^{\Delta B;\ L286R}$. Percent fusions were calculated as total number of chromosome fusions divided by total number of chromosomes scored. (**f**) Time course of the types of chromosome fusions observed in WT MEFs reconstituted with $TRF2^{\Delta B;\ L286R}$ after various length of times. sh*Trf2* was used to deplete endogenous TRF2. (i) 48h: metaphases with less than 1 telomere-free fusion/metaphase and <20% telomere-free chromosome ends; (ii) 72h: metaphase with ~2 telomere-free fusions/metaphase and ~50% telomere-free chromosome ends; (iii) 96h: metaphase with 3-5 telomere-free fusions/metaphase and >80% telomere-free chromosome ends; (iv) 120h: metaphase with >10 telomere-free chromosome fusions and ≤90% telomere-free chromosome ends. Arrows point to fusion sites devoid of telomeres. (**g**) Genomic DNA from WT MEFs expressing the indicated DNAs was fractionated with a clamped homogenous electric field (CHEF) gel, then in-gel hybridization was performed using a $(CCCTAA)_4$ probe to detect the 3' single-stranded (SS) overhang under native conditions (left) and under denaturing condition to detect total TTAGGG repeats (right). Single strand G overhang and total telomere signals in sh-control vector was set at 100% after normalizing each lane with ethidium bromide staining (left panel) that served as an internal loading control. For quantification, we used signals from the portions of the gel immediately below the wells to the 15 kb mark. The numbers below the gel represents normalized overhang values (middle panel) and total telomere signals (right panel) as compared to values from the sh-control vector. Molecular weights are displayed on the right.

h

Genotype	Metaphase scored	Total chromosome ends	Total signal free ends	% Signal free ends
Wild Type MEF +sh-control vector	25	4000	36	0.9
Wild Type MEF +sh*Trf2*+vector	30	4800	76	1.5
Wild Type MEF +sh*Trf2*+TRF2$^{\Delta B}$	40	6400	1400	22
Wild Type MEF +sh*Trf2*+TRF2$^{\Delta B;L286R}$	36	5760	3040	52.7
Rap1$^{-/-}$+sh-control vector	18	2880	95	3.2
Rap1$^{-/-}$+sh*Trf2*+vector	24	3840	176	4.1
Rap1$^{-/-}$+sh*Trf2*+TRF2$^{\Delta B}$	47	7520	3492	46

i

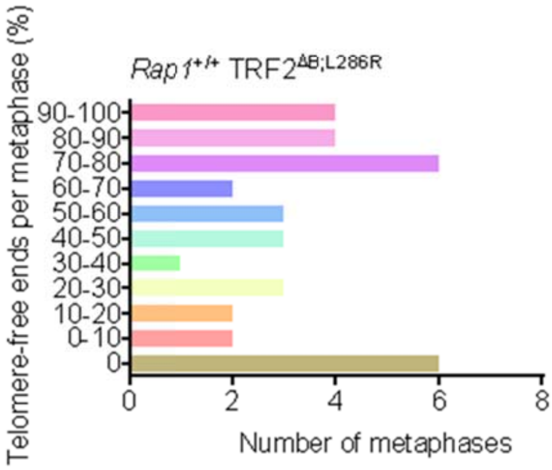

j

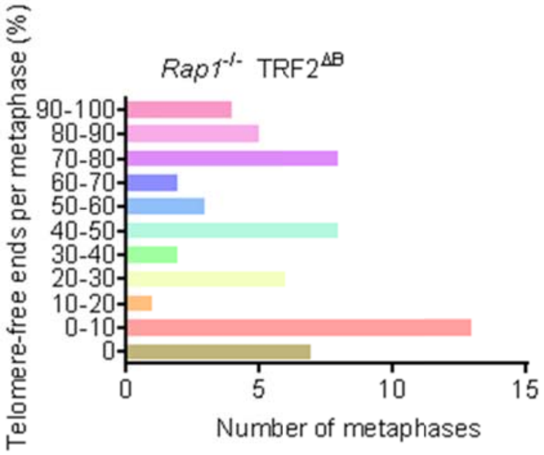

k

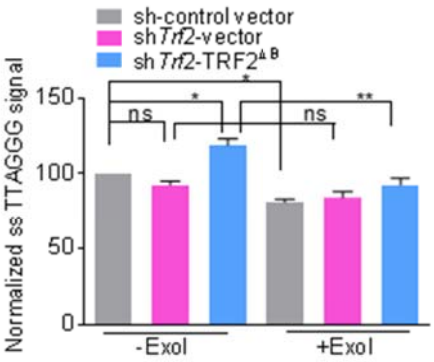

l

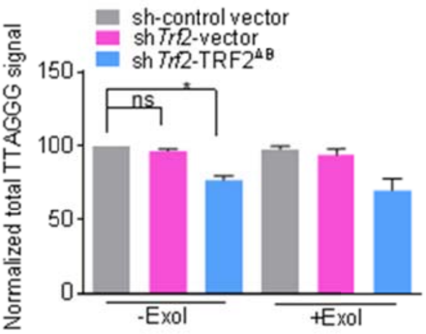

Supplementary Fig. 1. (**h**) Quantification of the percentage of telomere-free chromosome ends (signal-free ends) in metaphase spreads isolated from *Rap1*$^{+/+}$ or *Rap1*$^{-/-}$ MEFs reconstituted with either TRF2$^{\Delta B}$ or TRF2$^{\Delta B;\ L286R}$, followed by shRNA-mediated depletion of endogenous TRF2. (**i)** A total of 36 metaphases from *Rap1*$^{+/+}$ MEFs expressing TRF2$^{\Delta B;\ L286R}$ were scored for the percentage of chromosome ends without telomeres. Number of metaphases containing binned percent telomere-free ends (0-10%, 10-20%, etc) are shown. (**j**) A total of 47 metaphases from *Rap1*$^{-/-}$ MEFs expressing TRF2$^{\Delta B}$ were scored for the percentage of chromosome ends without telomeres. Number of metaphases containing 0-10%, 10-20%, etc telomere-free ends are shown. (**k**) Quantification of the amount of single-stranded telomeric signal shown in Fig. 1f (left panel). (**l**) Quantification of total telomeric signal shown in Fig. 1f (right panel). For (**k**) and (**l**), the single stranded G overhang and total telomere signals in sh-control vector was set at 100% after normalizing the lanes with ethidium bromide staining and to a subtelomeric fragment that served as an internal loading control. For quantification, we used signals from the portions of the gel immediately below the wells to the 15 kb mark. Data represent the mean of two experiments ±SD (*p<0.04; **p<0.08; one-way Anova). NS: not significant.

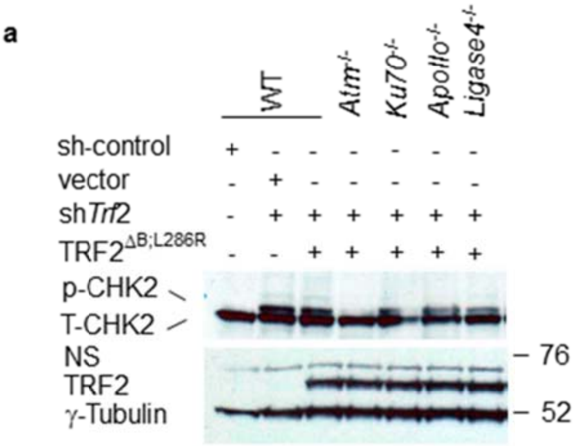

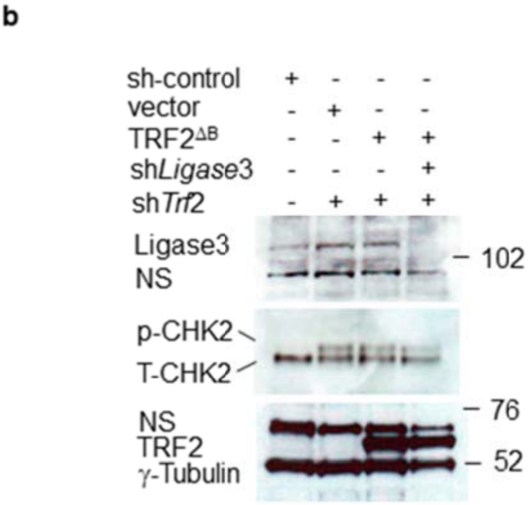

Supplementary Figure 2. (**a**) Immunoblots for TRF2, (T)-total CHK2 and (p) phosphorylated-CHK2 in MEFs of the indicated genotypes reconstituted with TRF2$^{\Delta B;\ L286R}$ followed by depletion of endogenous TRF2 with sh*Trf2*. γ-Tubulin was used a loading control. NS denotes the non-specific band above the TRF2 band. (**b**) Immunoblots for Ligase 3, TRF2, T-CHK2 and p-CHK2 in *Rap1*$^{-/-}$ MEFs expressing the indicated DNAs, in which TRF2$^{\Delta B}$ was first reconstituted and then endogenous TRF2 depleted with sh*TRF2*. γ-Tubulin used as loading control. NS denotes nonspecific band above the TRF2 and below the Ligase 3 bands. Note that our anti-TRF2 antibody cannot detect endogenous TRF2.

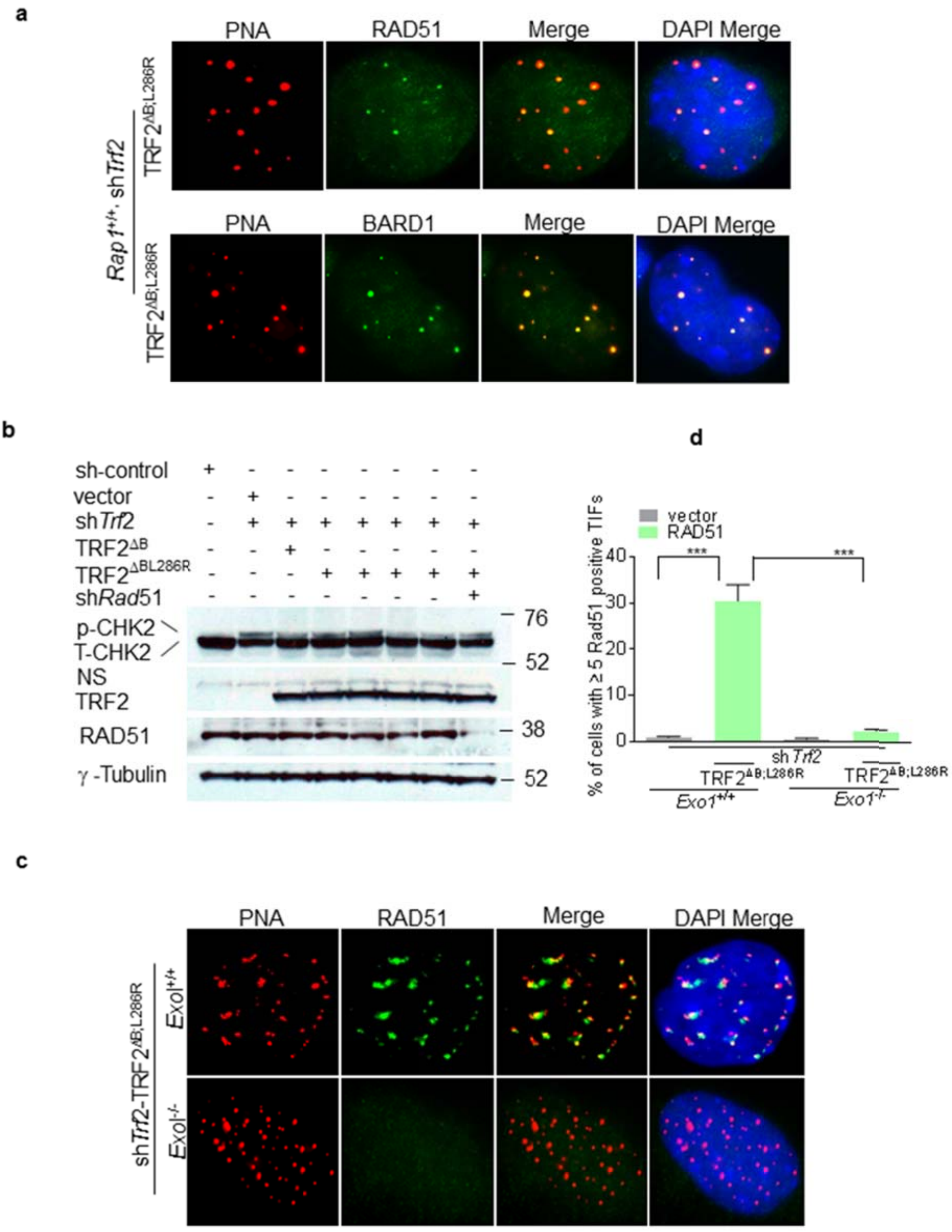

a
PNA
RAD51
Merge
DAPI Merge
BARD1
Rap1+/+, shTrf2
TRF2ΔB;L286R
b
sh-control
vector
shTrf2
TRF2ΔB
TRF2ΔBL286R
shRad51
p-CHK2
T-CHK2
NS
TRF2
RAD51
γ-Tubulin
76
52
38
52
c
shTrf2-TRF2ΔB;L286R
ExoI+/+
ExoI-/-
d
vector
RAD51
***
% of cells with ≥ 5 Rad51 positive TIFs
40
30
20
10
0
shTrf2
TRF2ΔB;L286R
Exo1+/+
Exo1-/-

Supplementary Figure 3. (**a**) Telomeric localization of RAD51 and BARD1 in WT MEFs reconstituted with TRF2$^{\Delta B;\ L286R}$ following depletion of endogenous TRF2 with *shTRF2*. Telomeres were visualized with PNA-FISH (red), anti-RAD51 or anti-BARD1 antibodies to visualize RAD51 or BARD1, respectively (green), and DAPI to visualize nuclei (blue). (**b**) Immunoblot to detect TRF2, RAD51, total (T) and phosphorylated (p) CHK2 in WT MEFs expressing the indicated DNAs followed by depletion of endogenous TRF2 with sh*TRF2***.** γ-Tubulin was used a loading control. NS denotes nonspecific band above TRF2 band. Note that our anti-TRF2 antibody cannot detect endogenous TRF2. (**c**) Localization of RAD51 to telomeres in *Exo1*$^{+/+}$ and *Exo1*$^{-/-}$ MEFs expressing TRF2$^{\Delta B;\ L286R}$. Telomeres were visualized with PNA-FISH (red), anti-RAD51 antibody (green) and DAPI to stain nuclei (blue). (**d**) Quantification of percent of cells with ≥ 5 RAD51 positive TIFs shown in (**c**). Data represents the mean ±SD from two independent experiments. A minimum of 75 nuclei were scored per experiments (***p<0.0003; one-way Anova).

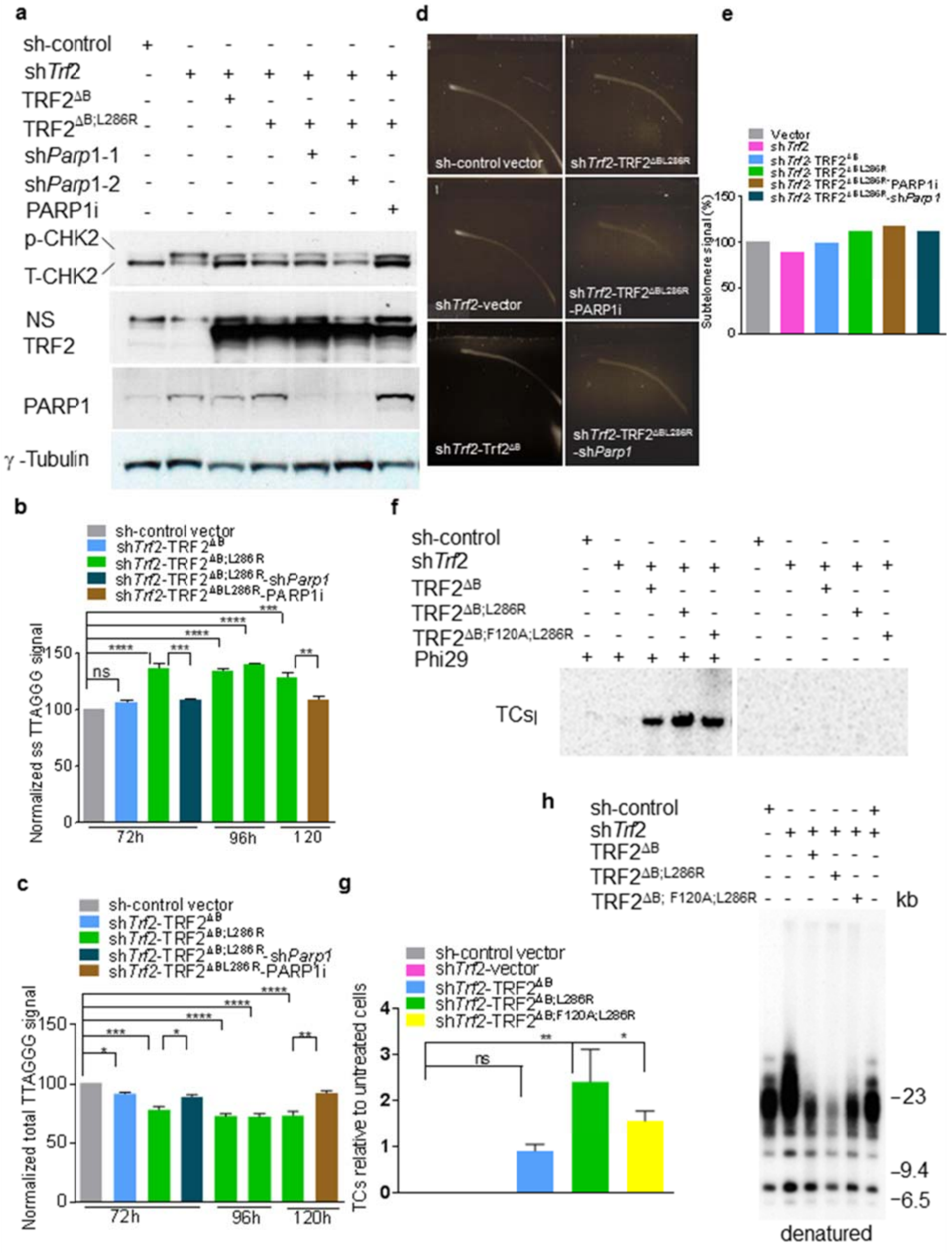
a
sh-control
shTrf2
TRF2ΔB
TRF2ΔB;L286R
shParp1-1
shParp1-2
PARP1i
p-CHK2
T-CHK2
NS
TRF2
PARP1
γ-Tubulin
b
sh-control vector
shTrf2-TRF2ΔB
shTrf2-TRF2ΔB;L286R
shTrf2-TRF2ΔB;L286R-shParp1
shTrf2-TRF2ΔBL286R-PARP1i
Normalized ss TTAGGG signal
ns
72h
96h
120
c
sh-control vector
shTrf2-TRF2ΔB
shTrf2-TRF2ΔB;L286R
shTrf2-TRF2ΔB;L286R-shParp1
shTrf2-TRF2ΔBL286R-PARP1i
Normalized total TTAGGG signal
72h
96h
120h
d
sh-control vector
shTrf2-TRF2ΔBL286R
shTrf2-vector
shTrf2-TRF2ΔBL286R -PARP1i
shTrf2-Trf2ΔB
shTrf2-TRF2ΔBL286R -shParp1
e
Vector
shTrf2
shTrf2-TRF2ΔB
shTrf2-TRF2ΔBL286R
shTrf2-TRF2ΔBL286R-PARP1i
shTrf2-TRF2ΔBL286R-shParp1
Subtelomere signal (%)
f
sh-control
shTrf2
TRF2ΔB
TRF2ΔB;L286R
TRF2ΔB;F120A;L286R
Phi29
TCs
g
sh-control vector
shTrf2-vector
shTrf2-TRF2ΔB
shTrf2-TRF2ΔB;L286R
shTrf2-TRF2ΔB;F120A;L286R
TCs relative to untreated cells
ns
h
sh-control
shTrf2
TRF2ΔB
TRF2ΔB;L286R
TRF2ΔB; F120A;L286R
kb
-23
-9.4
-6.5
denatured

Supplementary Figure 4. (**a**) Immunoblot for total CHK2, p-CHK2, TRF2 and PARP1 in *Ku70*$^{-/-}$ MEFs reconstituted with the indicated DNA constructs and subsequent removal of endogenous TRF2 by sh*TRF2*. In some experiments, 0.5 μM PARP1i or shRNA against *Parp1* was used to deplete endogenous PARP1. γ-Tubulin was used a loading control. NS denotes the nonspecific band above TRF2 band. Note that our anti-TRF2 antibody cannot detect endogenous TRF2. (**b**) Quantification of single stranded telomeric signal shown in Fig. 4f (left panel). Data represent the mean of two experiments ±SD. (**p<0.005, ***p<0.006, ****p<0.0001; one-way Anova). (**c**) Quantification of total telomeric signal shown in Fig. 4f (right panel). Data represent the mean of two experiments ±SD. (*p<0.03, **p<0.001, ***p<0.0004, ****p<0.0001; one-way Anova). Single strand G overhang and total telomere signals in sh-control vector was set at 100% after normalizing the lanes with both ethidium bromide staining and to a subtelomeric fragment that served as an internal control for loading. For quantification, we used signals from the portions of the gel immediately below the wells and up to the 15 kb mark. (**d**) Ethidium bromide staining of the 2D gels shown in Fig. 4g, revealing that the DNA loaded is approximately equal. **(e)**. Quantification of the intensities of the subtelomere repeat in linearized telomeres shown in Fig 4g, indicating that the samples are loaded equally. (**f**) Phi 29 dependent T-circle formation in WT MEFs expressing either TRF2$^{\Delta B}$ or TRF2$^{\Delta B;\ L286R}$. (**g**) Quantification of Phi 29 dependent T-circle formation in (**f**). Data represent the mean of two experiments ±SD. (*p<0.03, **p<0.005; one-way Anova). ns: non-significant values. (**h**) Genomic DNA from WT MEFs expressing the indicated DNAs was fractionated with a clamped homogenous electric field (CHEF) gel, and in-gel hybridization was performed after denaturing the gel using a $(CCCTAA)_4$ probe to detect total telomeric repeats. Molecular weights are displayed on the right.

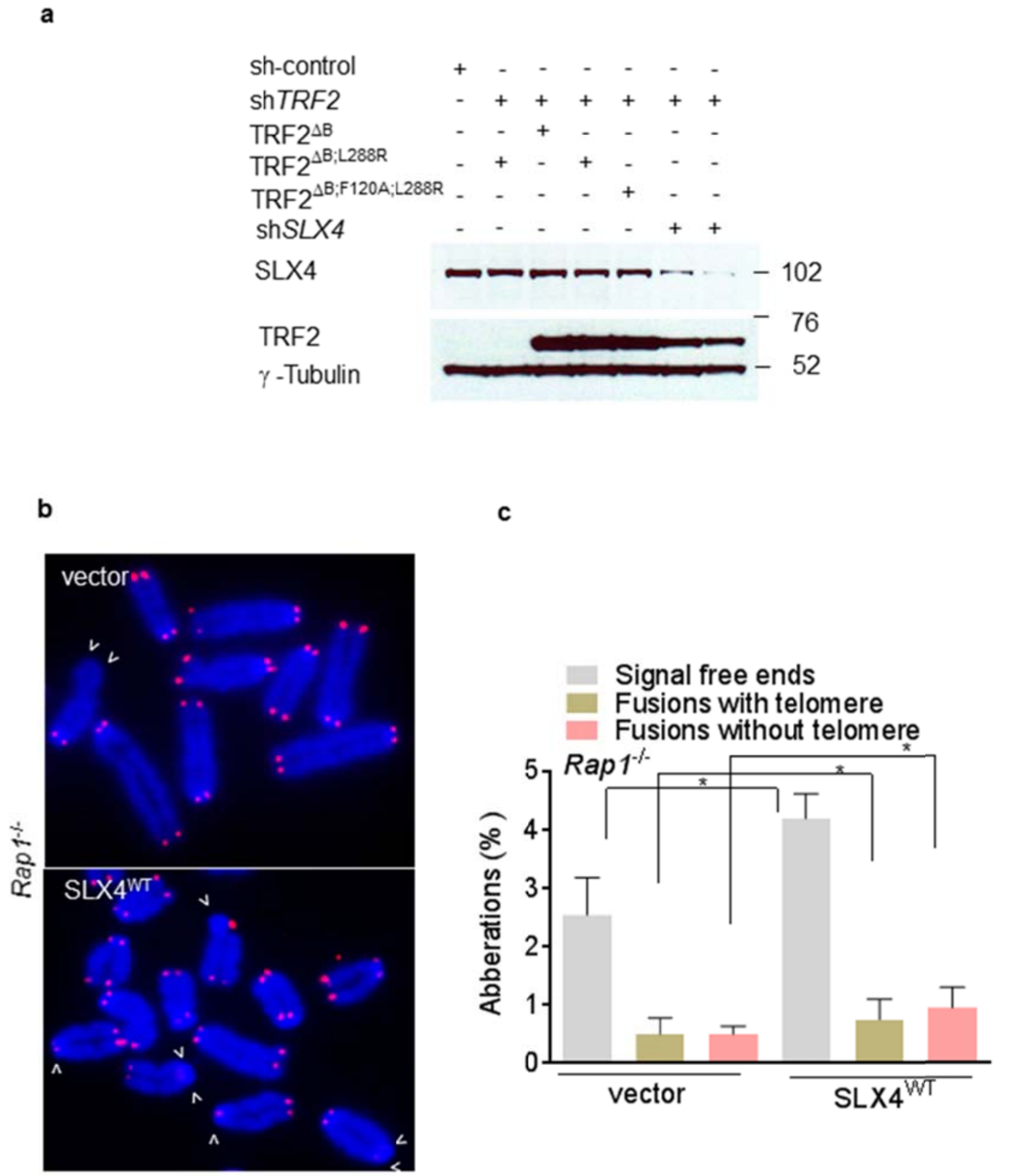

Supplementary Figure 5. (**a**) Immunoblots to detect SLX4 and TRF2 in IMR90 cells expressing the indicated DNAs followed by depletion of endogenous TRF2 with sh*TRF2*. γ-Tubulin was used a loading control. Note that our anti-TRF2 antibody cannot detect endogenous TRF2. (**b**) Partial metaphase spreads from *Rap1*$^{-/-}$ MEFs expressing either vector or mouse WT SLX4, visualized by telomere PNA-FISH (red) and DAPI (blue). Arrows point to signal-free ends. (**c**) Quantification of telomere signal free ends from representative images shown in (**b**). Data represent the mean of two experiments ±SD. (*p<0.02, one-way Anova).

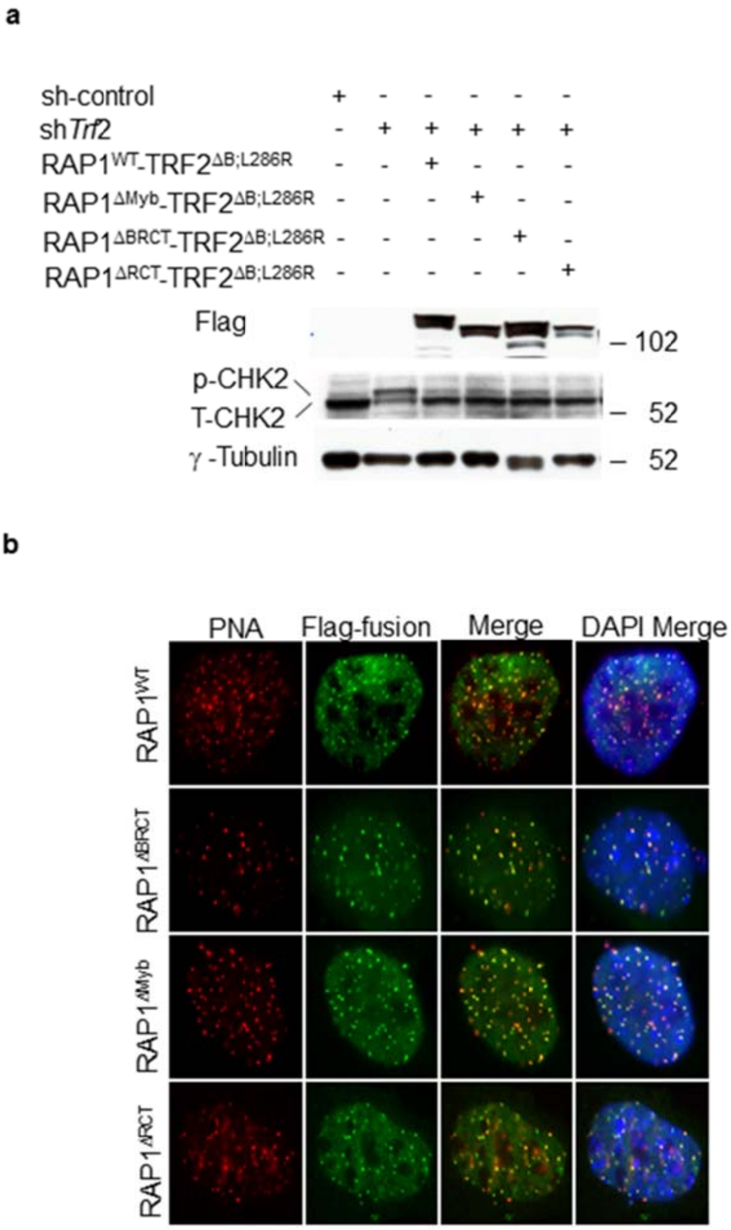

Supplementary Figure 6. (**a**) Immunoblots to detect the indicated Flag-tagged RAP1-TRF2 constructs, total CHK2 and p-CHK2 in WT MEFs in which endogenous TRF2 was depleted with *Trf2* shRNA. γ-Tubulin was used a loading control. Note that our anti-TRF2 antibody cannot detect endogenous TRF2. (**b**) Flag-tagged RAP1^{WT}-TRF2 and RAP1^{mutant}-TRF2 proteins localize to telomeres. Telomeres were visualized by PNA-FISH (red), Flag-tagged proteins (green) and DAPI (blue).

Figures 1c

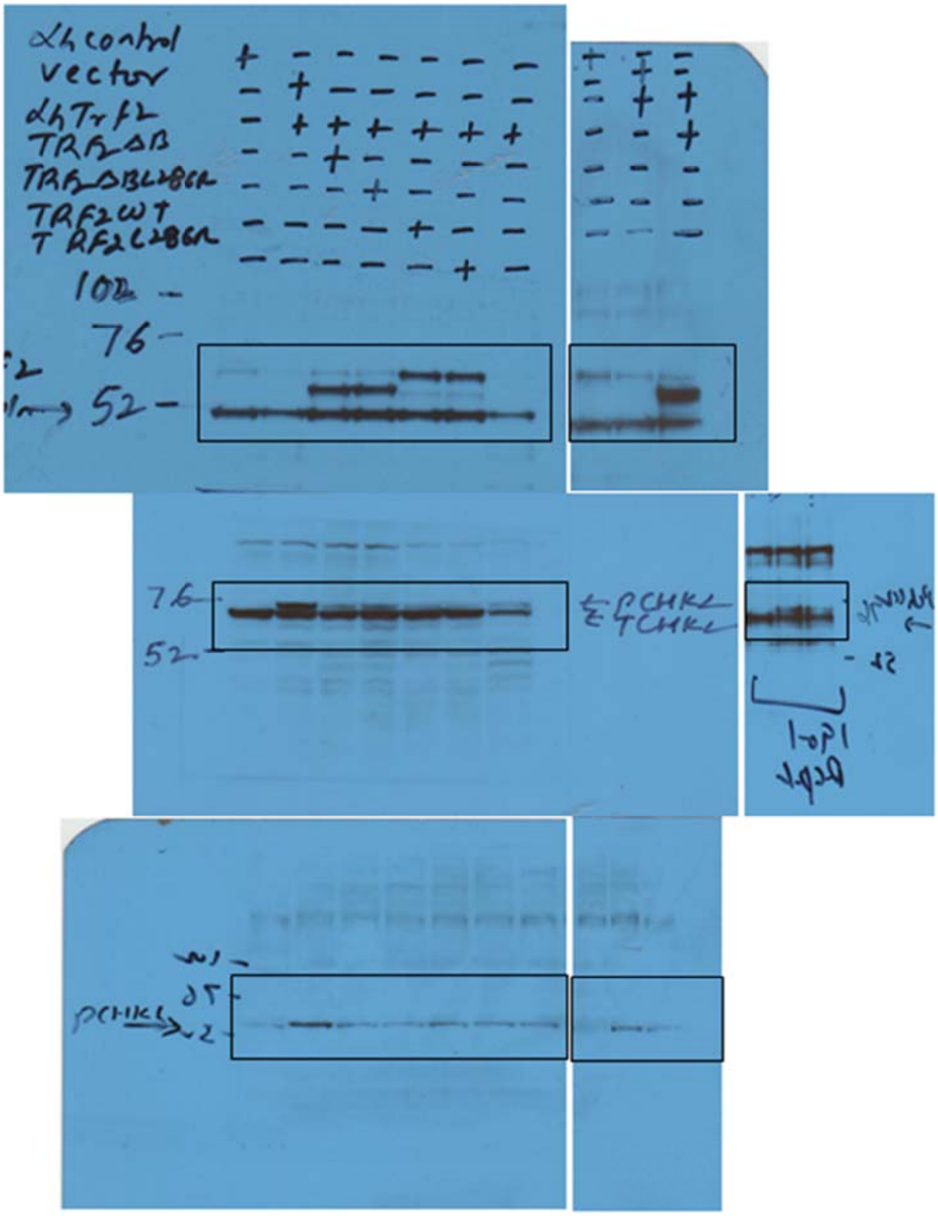

Supplementary Figure 7. Uncropped Western blots shown in figures and supplementary figures.

Supplementary Figure 2a

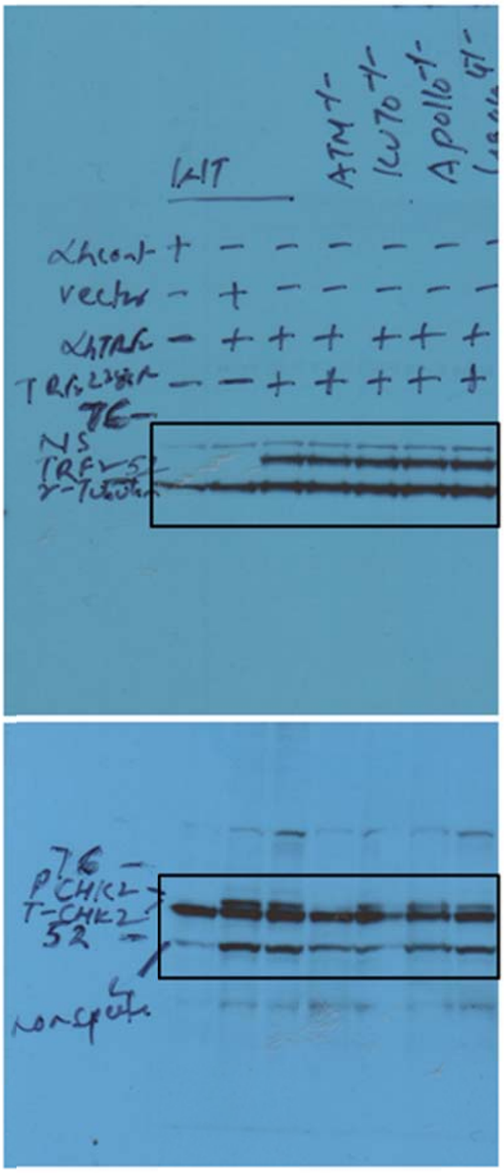

Supplementary Figure 2b

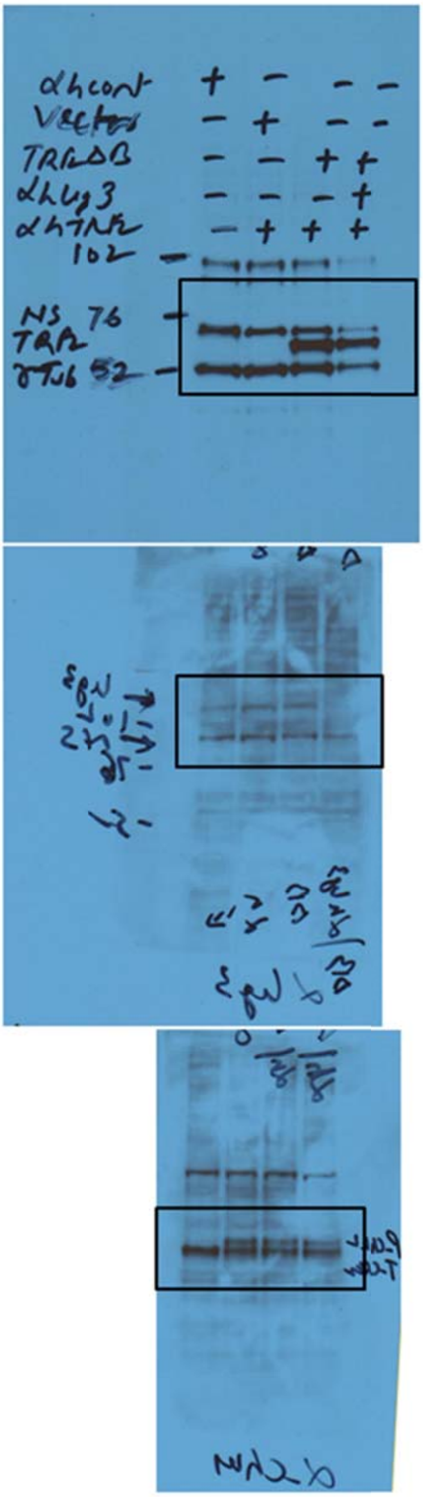

Supplementary Figure 7. Uncropped Western blots shown in figures and supplementary figures.

Supplementary Figure 3b

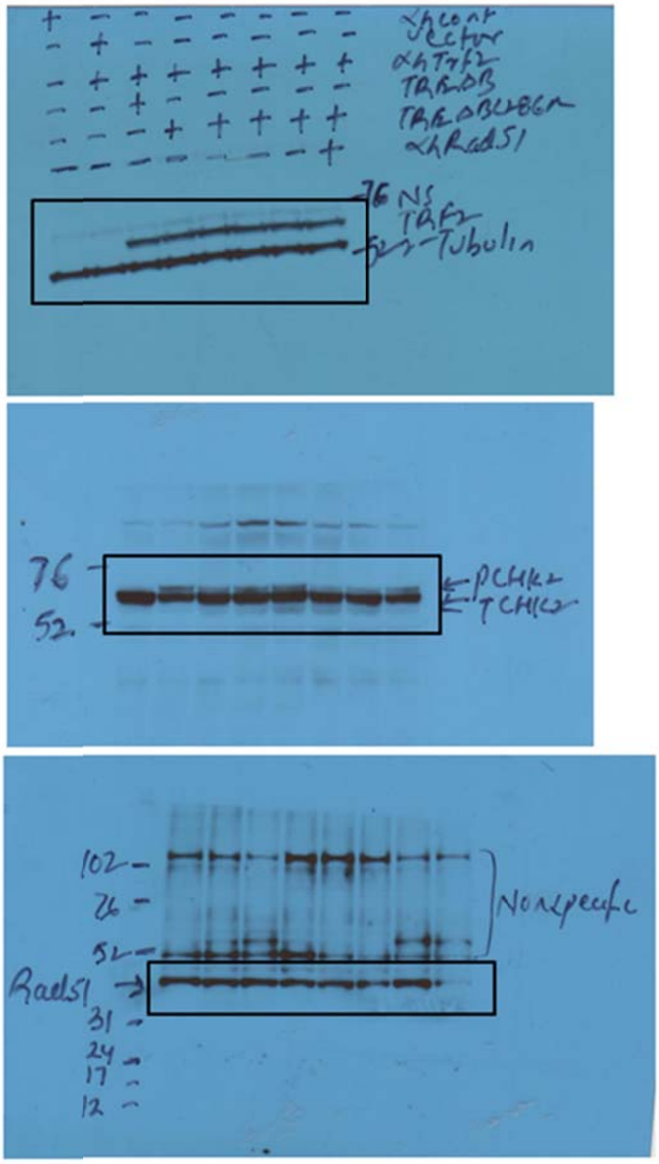

Supplementary Figure 4a

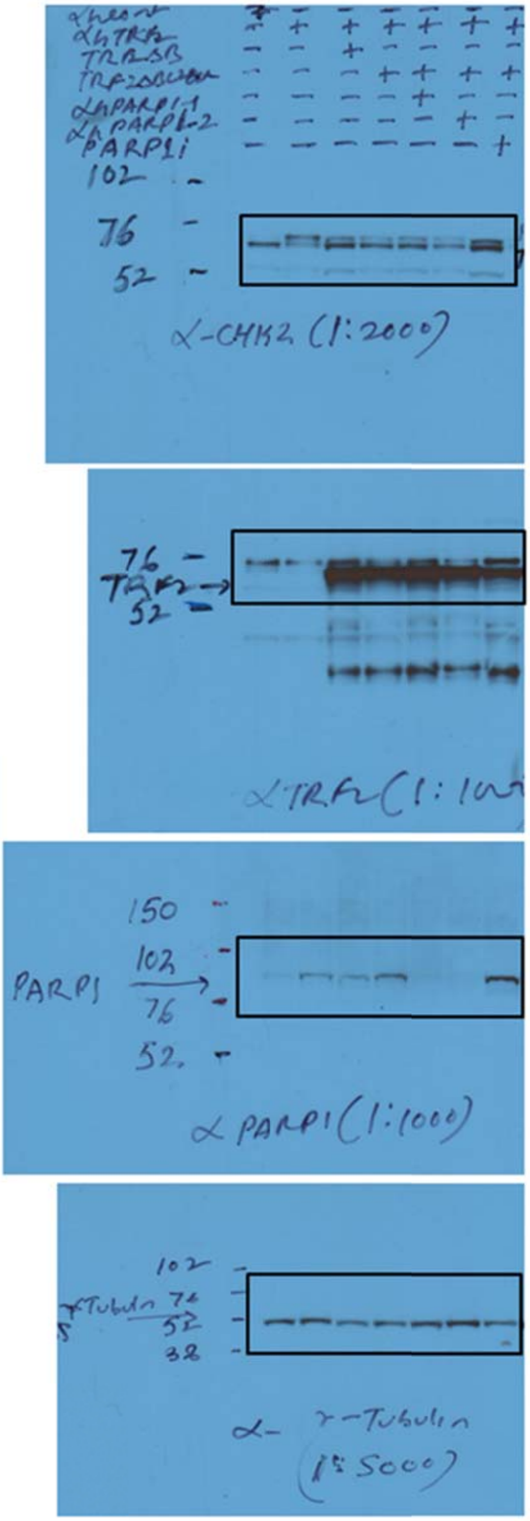

Supplementary Figure 7. Uncropped Western blots shown in figures and supplementary figures.

Supplementary Figure 5a

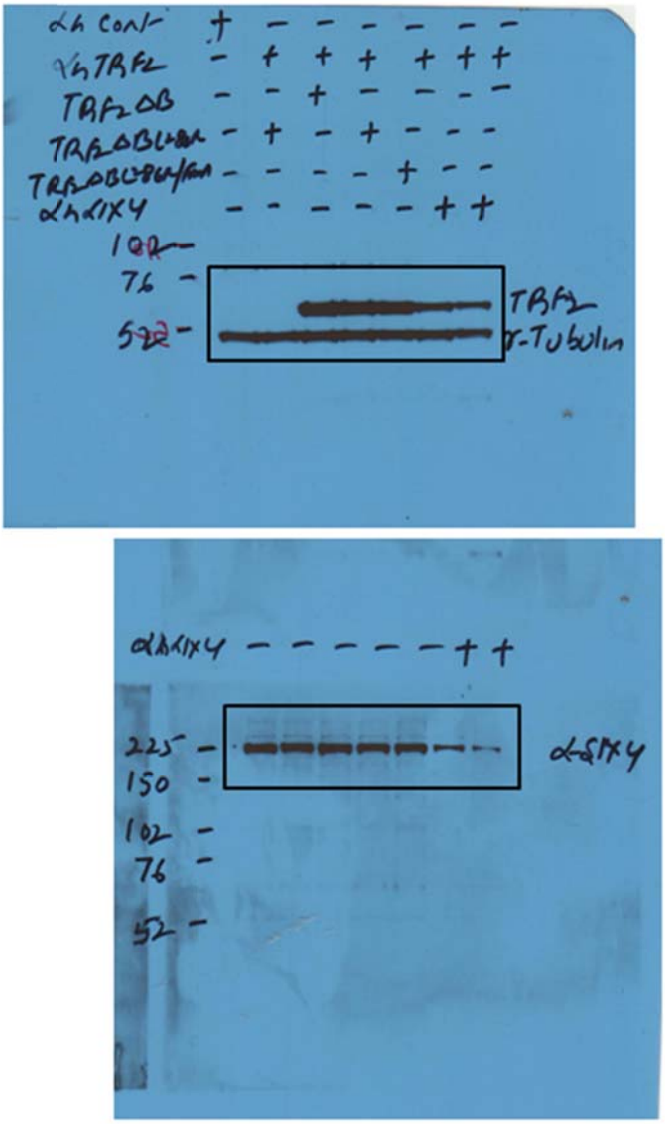

Supplementary Figure 6a

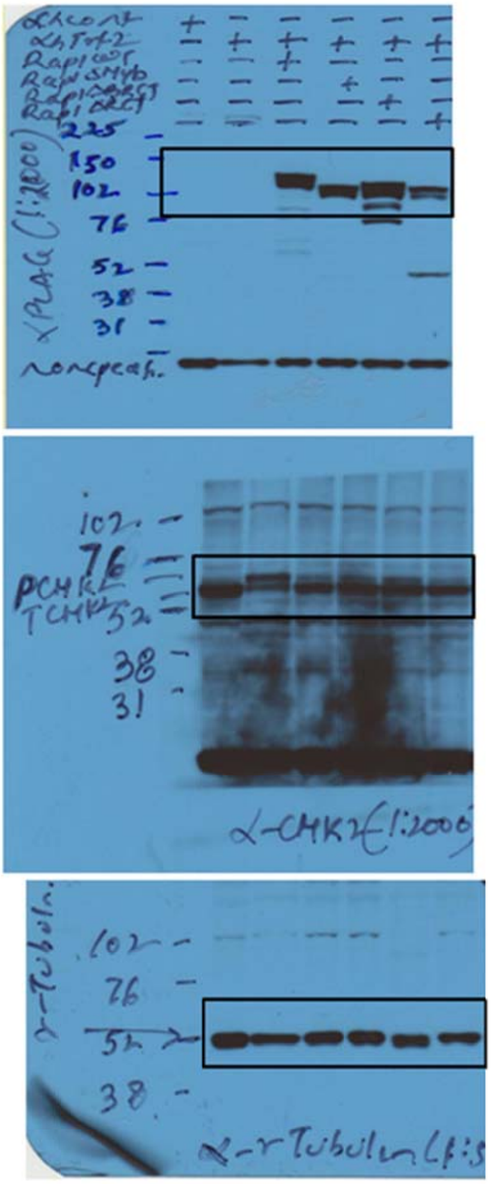

Supplementary Figure 7. Uncropped Western blots shown in figures and supplementary figures.
